# A TRPV4-dependent neuroimmune axis in the spinal cord promotes neuropathic pain

**DOI:** 10.1172/JCI161507

**Published:** 2023-03-01

**Authors:** Xueming Hu, Lixia Du, Shenbin Liu, Zhou Lan, Kaikai Zang, Jing Feng, Yonghui Zhao, Xingliang Yang, Zili Xie, Peter L. Wang, Aaron M. Ver Heul, Lvyi Chen, Vijay K. Samineni, Yan-Qing Wang, Kory J. Lavine, Robert W. Gereau, Gregory F. Wu, Hongzhen Hu

**Affiliations:** 1 Department of Anesthesiology, Center for the Study of Itch and Sensory Disorders, and Washington University Pain Center and; 2 Department of Pathology and Immunology, Washington University School of Medicine, St. Louis, Missouri, USA.; 3 Institute of Acupuncture and Moxibustion and Institute of Integrative Medicine; Department of Integrative Medicine and Neurobiology, School of Basic Medical Science; and State Key Laboratory of Medical Neurobiology, Institutes of Brain Science, Fudan University, Shanghai, China.; 4 Department of Internal Medicine, Cardiovascular Division and; 5 Department of Neurology, Washington University School of Medicine, St. Louis, Missouri, USA.

**Keywords:** Neuroscience, Pain

## Abstract

Microglia, resident macrophages of the CNS, are essential to brain development, homeostasis, and disease. Microglial activation and proliferation are hallmarks of many CNS diseases, including neuropathic pain. However, molecular mechanisms that govern the spinal neuroimmune axis in the setting of neuropathic pain remain incompletely understood. Here, we show that genetic ablation or pharmacological blockade of transient receptor potential vanilloid type 4 (TRPV4) markedly attenuated neuropathic pain-like behaviors in a mouse model of spared nerve injury. Mechanistically, microglia-expressed TRPV4 mediated microglial activation and proliferation and promoted functional and structural plasticity of excitatory spinal neurons through release of lipocalin-2. Our results suggest that microglial TRPV4 channels reside at the center of the neuroimmune axis in the spinal cord, which transforms peripheral nerve injury into central sensitization and neuropathic pain, thereby identifying TRPV4 as a potential new target for the treatment of chronic pain.

## Graphical abstract

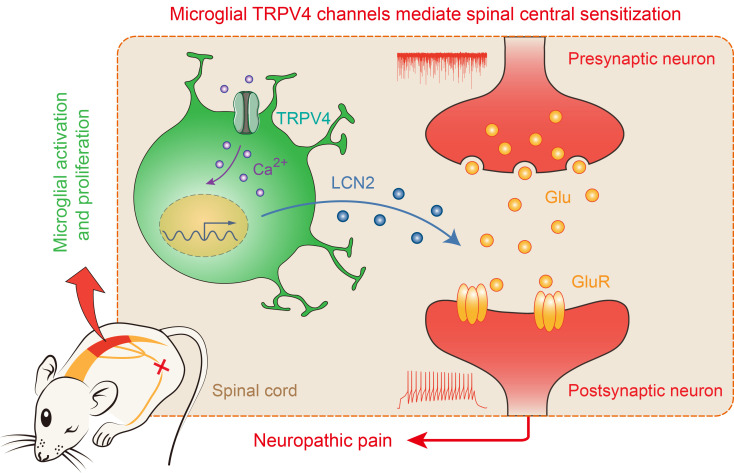

## Introduction

Whereas acute pain is a protective mechanism to avoid further damage, chronic pain is a maladaptive, debilitating condition affecting hundreds of millions of people worldwide (1, 2). About a fifth of people who have chronic pain predominantly have neuropathic pain as result of tissue or nerve injury (3). Although several drugs for relieving chronic pain are available, the use of current first-line pain medicines such as nonsteroidal antiinflammatory drugs, antidepressants, and opioids is limited by serious side effects, dependence, tolerance, and insufficient efficacy (4, 5). This has encouraged major efforts in both academia and industry to develop safer and more effective pain therapies. Notably, current treatments for chronic pain mainly focus on blocking neurotransmission in the pain pathway and have resulted only in limited success. Ironically, although nerve injury produces robust microgliosis, therapeutic strategies targeting microglia for chronic pain treatment remain largely overlooked.

Microglia, tissue-resident macrophages in the CNS, perform dynamic surveillance of their microenvironment to maintain tissue homeostasis. However, when exposed to pathological stimuli, microglia conversely change their functional phenotypes and secrete an excess of proinflammatory cytokines and reactive oxygen species, resulting in maladaptive neurological disorders (6). For instance, following peripheral nerve injury, microglia in the spinal cord convert injury signals from primary sensory neurons, leading to persistent central sensitization and chronic neuropathic pain (7, 8). However, although it has emerged as a vital event for the transition from acute to chronic pain state (9), the molecular mechanisms underlying the interactions between microglia and pain-transmitting spinal neurons are still poorly understood. Specifically, 2 key questions remain to be fully addressed: what is the molecular basis of microgliosis driven by peripheral nerve injury and how does microglial activation results in neuroplasticity leading to chronic neuropathic pain?

The transient receptor potential (TRP) channels are a group of Ca^2+^-permeable nonselective cation channels that are indispensable for transmitting a wide range of somatosensory stimuli, including itch, pain, temperature, and mechanosensation. Thus, they are typically considered molecular sensors in the primary sensory afferent nociceptors (10). Surprisingly, emerging evidence suggests that TRP channels are also widely expressed and have many physiological and pathological functions in both neurons and nonneuronal cells (11, 12). We and others have recently demonstrated that TRP vanilloid type 4 (TRPV4) expressed by macrophages and microglia is associated with diverse pathophysiological processes (13–15).

Here, we show that TRPV4 was functionally expressed in spinal resident microglia and TRPV4 expression was increased in a mouse model of spared nerve injury (SNI). Genetic ablation and pharmacological inhibition of microglia-expressed TRPV4 markedly suppressed SNI-induced neuropathic pain-related behaviors. Mechanistically, TRPV4 mediated microglial activation and proliferation and promoted functional and structural plasticity of excitatory spinal neurons by releasing lipocalin-2 (LCN2) from activated microglia after SNI. Collectively, our data identified the TRPV4-dependent neuroimmune axis as an essential component required for neuropathic pain generation and provided potential drug targets for the treatment of chronic neuropathic pain with decreased reliance on opioids.

## Results

### Functional expression of TRPV4 in spinal cord microglia.

**Figure 1:**
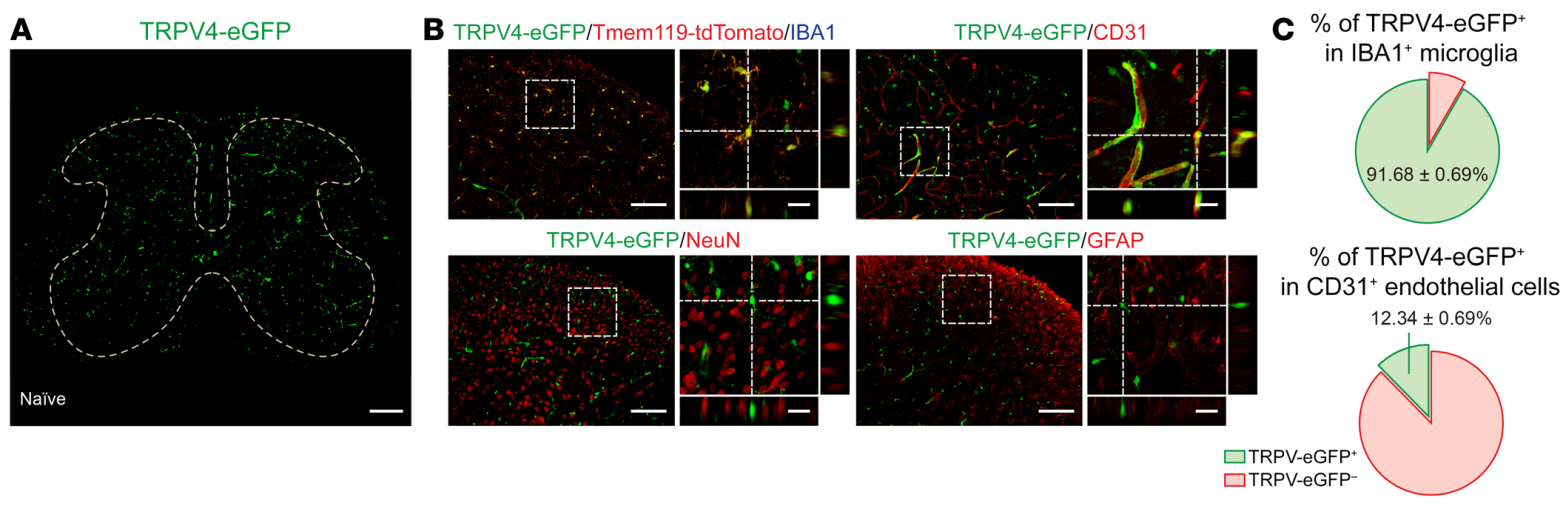
TRPV4 is expressed by spinal microglia. (**A**) Representative image of TRPV4-eGFP expression in the spinal cord of naive mice. Scale bar: 200 μm. (**B** and **C**) Representative images of TRPV4-expressing cells (TRPV4-eGFP^+^) with microglial marker IBA1, endothelial marker CD31, neuronal marker NeuN, and astrocytic marker GFAP in the spinal dorsal horn of naive mice (**B**), and quantification of the proportion of TRPV4-eGFP^+^/IBA1^+^ microglia and TRPV4-eGFP^+^/ CD31^+^ endothelial cells. *n* = 10 spinal slices from 3 mice (**C**). (**B** and **C**) Scale bar: 100 μm and 20 μm (zoom).

Although TRPV4 is traditionally believed to be functionally expressed by primary nociceptors (16–19), surprisingly, in the spinal cord, we found no TRPV4-eGFP expression in TRPV1^+^ primary–afferent terminals by using the *Trpv4^eGFP^*:*Trpv1^Cre/+^*:tdTomato reporter mice (Supplemental Figure 1A; supplemental material available online with this article; https://doi.org/10.1172/JCI161507DS1). Instead, we detected TRPV4-eGFP signals predominantly in IBA1^+^/Tmem119^+^ microglia and CD31^+^ endothelial cells (20), but not in NeuN^+^ neurons or GFAP^+^ astrocytes in the spinal cord (Figure 1, A–C, and Supplemental Figure 1B), suggesting that TRPV4 may participate in pain signaling through immunogenic instead of direct neurogenic mechanisms.

**Figure 2:**
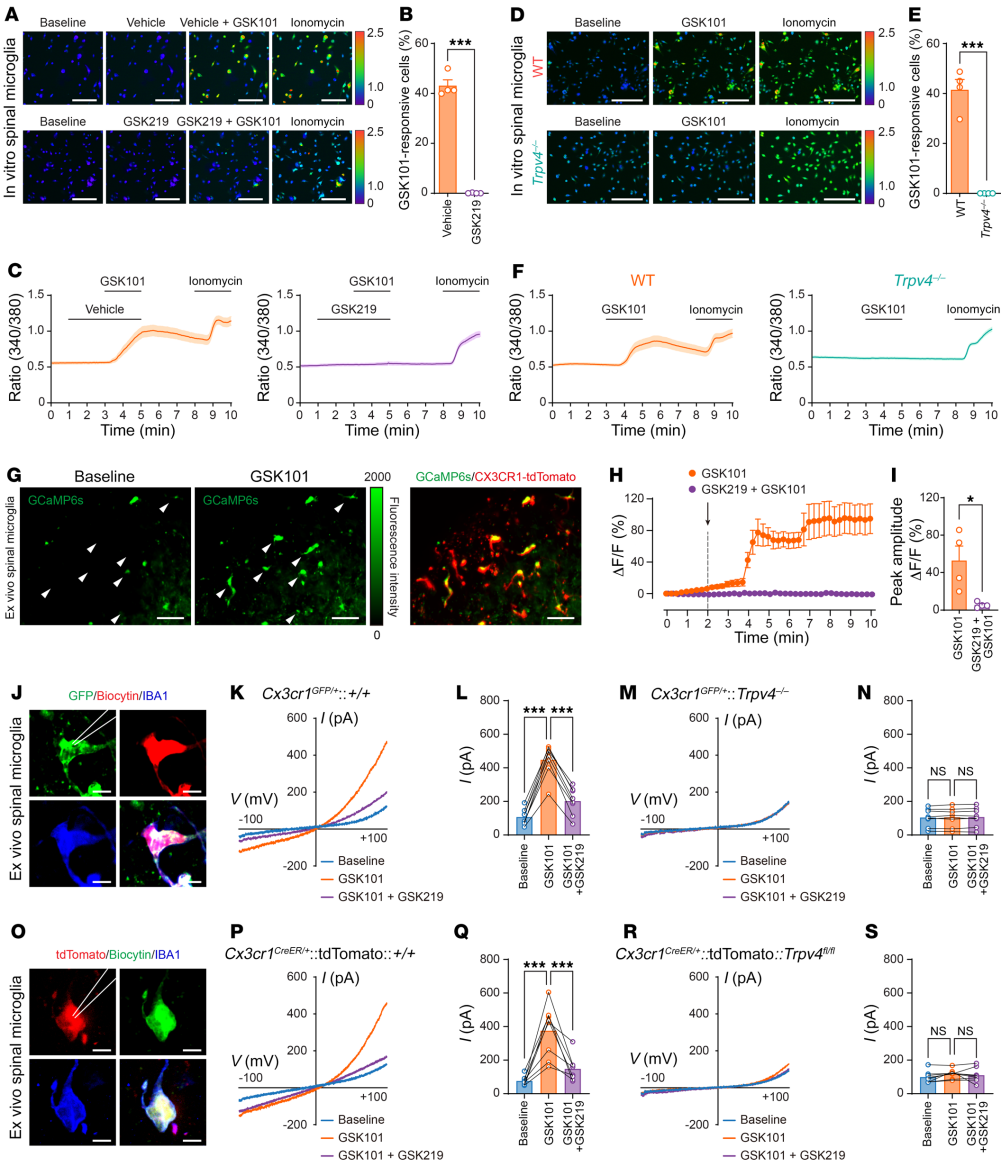
TRPV4 mediates [Ca^2+^]_i_ responses in spinal microglia. (**A**–**C**) Representative Ca^2+^ imaging (**A**), percentage (**B**), and time-lapse averaged Ca^2+^ traces (**C**) of in vitro responses to GSK101 (300 nM) and GSK219 (300 nM) exposure in primary spinal microglia from WT mice. (**D**–**F**) Representative Ca^2+^ imaging (**D**), percentage (**E**), and time-lapse averaged Ca^2+^ traces (**F**) of in vitro responses to GSK101 (300 nM) exposure in primary spinal microglia from WT and *Trpv4^–/–^* mice. Ionomycin (1 μM) was used as a positive control. *n* = 4 coverslips per group from 2 independent experiments. ***P < 0.001 by unpaired 2-tailed Student’s *t* test. Scale bar: 50 μm. (**G**–**I**) Representative Ca^2+^ imaging (**G**), time-lapse averaged Ca^2+^ traces (**H**), and Δ*F*/*F* peak amplitude (**I**) of ex vivo responses to GSK101 (300 nM) and GSK219 (300 nM) exposure in spinal microglia from *Cx3cr1^CreER/+^*:tdTomato:GCaMP6s mice. *n* = 4 mice per group. Scale bar: 50 μm. *P < 0.05 by unpaired 2-tailed Student’s *t* test. (**J**–**N**) GSK101-activated whole-cell membrane currents in spinal GFP^+^ microglia. Representative recorded GFP^+^ microglia images. Scale bar: 10 μm (**J**). Current-voltage curves and quantification of GSK101 (300 nM) and GSK219 (300 nM) on currents recorded at +100 mV from *Cx3cr1^GFP/+^* mice (**K** and **L**) and *Cx3cr1^GFP/+^*:*Trpv4^–/–^* mice (**M** and **N**). (**O**–**S**) GSK101-activated whole-cell membrane currents in spinal tdTomato^+^ microglia. Representative recorded tdTomato^+^ microglia images. Scale bar: 10 μm (**O**). Current-voltage curves and quantification of GSK101 (300 nM) and GSK219 (300 nM) on currents recorded at +100 mV from *Cx3cr1^CreER/+^*:tdTomato mice (**P** and **Q**) and *Cx3cr1^CreER/+^*:tdTomato:*Trpv4^fl/fl^* mice (**R** and **S**). *n* = 8 cells from 3 mice per group. ***P < 0.001 by 1-way ANOVA with Bonferroni’s post hoc test. Data are represented as mean ± SEM.

Given that TRPV4 is a Ca^2+^-permeable ion channel, we corroborated the observation of TRPV4 expression in spinal microglia using Ca^2+^ imaging with functional verification. We first performed in vitro Ca^2+^ imaging in cultured primary spinal microglia isolated from both newborn WT *C57BL/6J* and *Trpv4^–/–^* mice. Perfusion with a selective TRPV4 agonist GSK1016790A (GSK101) elicited robust intracellular Ca^2+^ ([Ca^2+^]_i_) responses, which were nearly abolished by coapplied TRPV4 antagonist GSK2193874 (GSK219, Figure 2, A–C). Consistent with pharmacological inhibition studies, GSK101 did not elicit any measurable [Ca^2+^]_i_ responses in the *Trpv4^–/–^* spinal microglia, while ionomycin, used as positive control, elicited robust [Ca^2+^]_i_ responses in all cells tested (Figure 2, D–F). To avoid potential issues related to the development and cell culture conditions using microglia from newborn mice, we further performed ex vivo Ca^2+^ imaging in acutely prepared spinal slices from adult *Cx3cr1^CreER/+^*:tdTomato:GCaMP6s mice (21). Perfusion with GSK101 elicited large [Ca^2+^]_i_ transients in the spinal dorsal horn tdTomato^+^ cells, which was abolished by coapplied GSK219 (Figure 2, G–I). Moreover, we directly recorded GSK101-activated whole-cell currents in ex vivo spinal dorsal horn microglia using the whole-cell patch-clamp recording. Acute perfusion with GSK101 activated robust outwardly rectifying TRPV4-like currents in the spinal microglia from both adult *Cx3cr1^GFP/+^* mice (Figure 2, J–L) and *Cx3cr1^CreER/+^*:tdTomato mice (Figure 2, O–Q), which were abolished by GSK219. Consistent with the pharmacological blockade experiments, the GSK101-activated TRPV4-like currents were completely absent in spinal microglia from adult *Cx3cr1^GFP/+^*:*Trpv4^–/–^* mice (Figure 2, M and N) or *Cx3cr1^CreER/+^*:tdTomato:*Trpv4^fl/fl^* mice (Figure 2, R and S). Collectively, these results suggest that microglia-expressed TRPV4 is the sole mediator of GSK101-induced responses.

### TRPV4 was required for pain-related behaviors following spared nerve injury.

**Figure 3:**
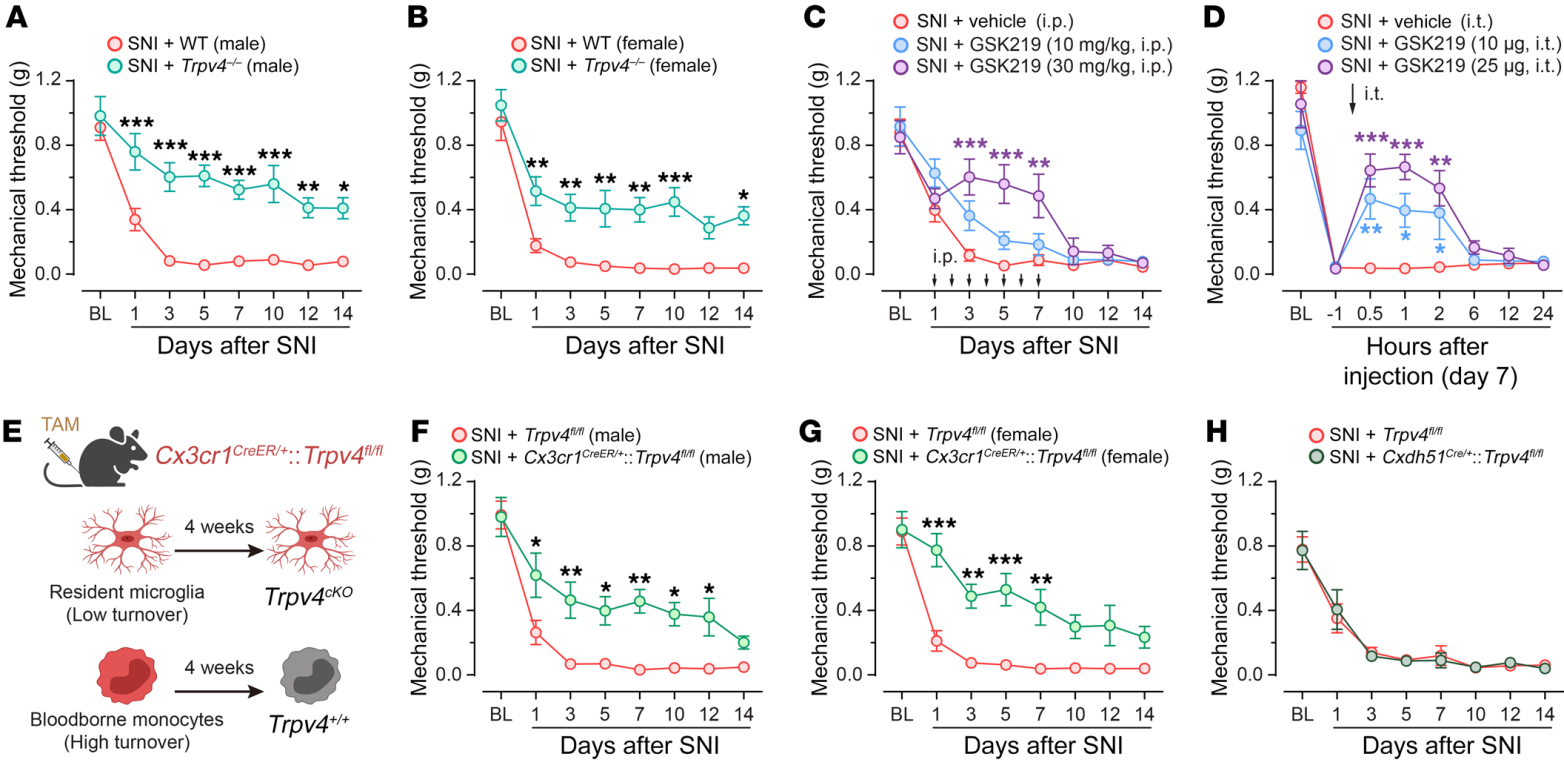
Genetic or pharmacological inhibition of TRPV4 suppresses neuropathic pain. (**A** and **B**) Time course of paw withdrawal threshold following SNI in male (**A**) and female (**B**) WT control littermates and *Trpv4^–/–^* mice. *n* = 7–10 mice per group. Statistics were determined by 2-way repeated ANOVA with Bonferroni’s post hoc test. (**C**) Time course of paw withdrawal threshold following SNI in WT mice treated with repeated i.p. injection of vehicle or GSK219 (once per day from day 1–7 after SNI). *n* = 7–10 mice per group. Statistics were determined by 2-way repeated ANOVA with Bonferroni’s post hoc test. (**D**) Time course of paw withdrawal threshold (von Frey) following SNI in WT mice treated with single i.t. injection of vehicle or GSK219 on day 7 after SNI. *n* = 5–8 mice per group. Statistics were determined by 2-way repeated ANOVA with Bonferroni’s post hoc test. (**E**) Schematic protocol (created with BioRender) for distinguishing between resident microglia and infiltrating monocytes using *Cx3cr1^CreER/+^* mice with tamoxifen (TAM) treatment for 5 consecutive days. 4 weeks after TAM-induced Cre-LoxP recombination, resident microglia are *Trpv4^cKO^* (*Cx3cr1^CreER/+^*:*Trpv4^fl/fl^*) while circulating monocytes are *Trpv4^+/+^*, dependent on the different turnover rate. (**F** and **G**) Time course of paw withdrawal threshold (von Frey) following SNI in male (**F**) and female (**G**) *Trpv4^fl/fl^* control littermates and *Cx3cr1^CreER/+^*:*Trpv4^fl/fl^* mice. *n* = 7–10 mice per group. Statistics were determined by 2-way repeated ANOVA with Bonferroni’s post hoc test. (**H**) Time course of paw withdrawal threshold (von Frey) following SNI in *Trpv4^fl/fl^* control littermates and *Cdh5^Cre/+^*:*Trpv4^fl/fl^* mice. *n* = 6–8 mice per group. Data are presented as mean ± SEM. *P < 0.05, **P < 0.01, ***P < 0.001.

Although sensory TRP channels are generally considered critical molecular sensors for mechanical, thermal, and chemical cues and contribute to both pain and itch sensations (10, 22–24), the role of centrally expressed TRPV4 in neuropathic pain remains poorly understood. To test this possibility, we generated a well-established mouse model of SNI (25) in both *Trpv4^–/–^* (global *Trpv4* KO) and congenic WT *C57BL/6J* mice. Strikingly, compared with WT mice, *Trpv4^–/–^* mice had a significantly improved paw withdrawal threshold, a hallmark of mechanical allodynia, in both male and female mice following SNI (Figure 3, A and B). To complement genetic ablation studies, we administered TRPV4 antagonist GSK219 once daily through repeated i.p. injections for 7 consecutive days starting on day 1 after SNI in WT mice and observed a marked increase in paw withdrawal threshold in a dose- and time-dependent manner (Figure 3C). Moreover, acute application of GSK219 through a single intrathecal (i.t.) injection on day 7 after SNI also produced a time- and dose-dependent attenuation of the decreased paw withdrawal threshold caused by SNI (Figure 3D), suggesting that TRPV4 in the spinal cord is involved in both initiation and maintenance of neuropathic pain. Note that i.p. or i.t. injections of GSK219 alone had no significant effect on the paw withdrawal threshold (Supplemental Figure 2, A and B). Next, we directly tested whether the expression of TRPV4 in spinal resident microglia contributed to neuropathic pain using inducible *Cx3cr1^CreER/+^*:*Trpv4^fl/fl^* (microglia-specific *Trpv4* cKO) mice 4 weeks after tamoxifen treatment. The SNI-induced mechanical allodynia was also attenuated in both male and female *Cx3cr1^CreER/+^*:*Trpv4^fl/fl^* mice, compared with *Trpv4^fl/fl^* control littermates (Figure 3, E–G). In marked contrast, mice ablating TRPV4 in either CDH5^+^ endothelial cells (*Cdh5^Cre/+^*:*Trpv4^fl/fl^* mice, Figure 3H), TRPV1^+^ primary sensory neurons (*Trpv1^Cre/+^*:*Trpv4^fl/fl^* mice, Supplemental Figure 3A), or CCR2^+^ blood-borne monocytes (*Ccr2^CreER/+^*:*Trpv4^fl/fl^* mice, Supplemental Figure 3B) exhibited no significant changes in SNI-induced mechanical allodynia.

To confirm the absence of functional TRPV4 in mouse spinal neurons, we selectively knocked down TRPV4 function in the spinal neurons by intraspinal injection of AAV-hSyn-EGFP-Cre viral vector in the *Trpv4^fl/fl^*:tdTomato mice. The AAV-hSyn-EGFP was used as a control viral vector (Supplemental Figure 4A). Three weeks after virus injection, the expression of EGFP and tdTomato signals were found to be expressed by the spinal dorsal horn NeuN^+^ neurons (Supplemental Figure 4B), but not by IBA1^+^ microglia, CD31^+^ endothelial cells, or GFAP^+^ astrocytes (Supplemental Figure 4, C–E), validating the specificity of the hSyn promotor and efficacy of Cre recombination. However, these neuron-specific *Trpv4* knockdown mice did not exhibit obvious changes in sensorimotor behaviors (Supplemental Figure 4, F and G) or improved mechanical hypersensitivity after the SNI procedure (Supplemental Figure 4H). These results ruled out a direct action of TRPV4 in the spinal neurons in SNI-induced pain hypersensitivity.

**Figure 4:**
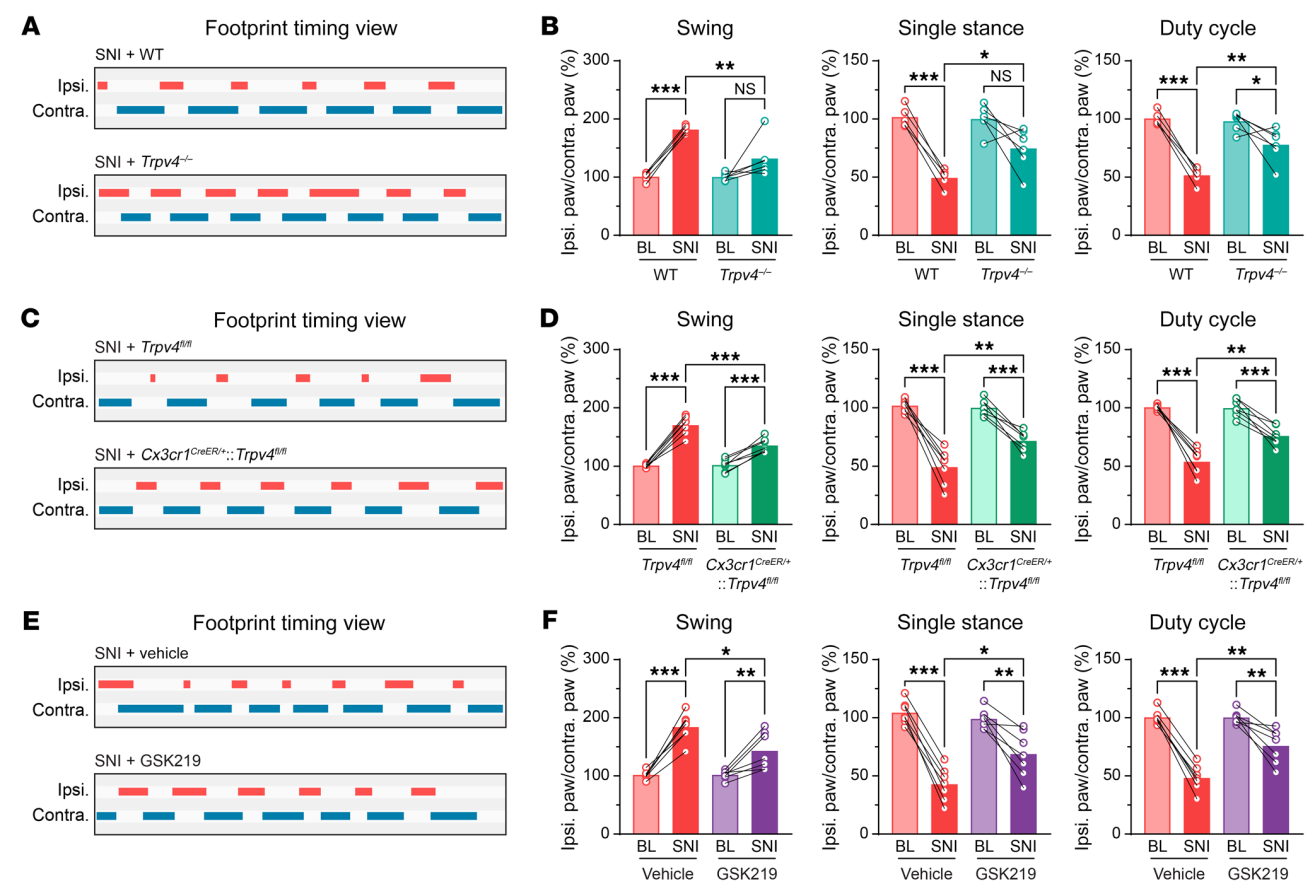
Genetic or pharmacological inhibition of TRPV4 suppresses abnormal gait following SNI. (**A** and **B**) Representative footprint timing view and CatWalk gait analysis of WT mice and *Trpv4^–/–^* mice on day 7 after SNI. *n* = 5–6 mice per group. Statistics were determined by paired or unpaired 2-tailed Student’s *t* test. (**C** and **D**) Representative footprint timing view and CatWalk gait analysis of *Trpv4^fl/fl^* control littermates and *Cx3cr1^CreER/+^*:*Trpv4^fl/fl^* mice on day 7 after SNI. *n* = 7 mice per group. Statistics were determined by paired or unpaired 2-tailed Student’s t test. (**E** and **F**) Representative footprint timing view and CatWalk gait analysis of WT mice treated with i.p. injection of vehicle or GSK219 (30 mg/kg, once per day from day 1–7 after SNI) on day 7 after SNI. *n* = 7 mice per group. Statistics were determined by paired or unpaired 2-tailed Student’s *t* test. Data are presented as mean ± SEM. *P < 0.05, **P < 0.01, ***P < 0.001.

Besides reflexive pain-related behaviors, we also performed CatWalk gait analysis in mice subjected to SNI. This quantitative gait analysis represents an objective way to reflect changes in various walking parameters due to pain and motor impairment and has been successfully used in previous studies to assess nonreflexive pain behaviors in animal models (26). As predicted, the percentage of swing was increased, whereas the percentages of single stance and duty cycle were decreased on day 7 after SNI, which could be representative of pain-like behaviors from weight-bearing and limb-using aspects (27). Importantly, similar to reflexive pain-related behaviors, these SNI-induced gait alterations were also significantly improved in the *Trpv4^–/–^* mice (Figure 4, A and B), *Cx3cr1^CreER/+^*:*Trpv4^fl/fl^* mice (Figure 4, C and D), or in WT mice treated with repeated i.p. injections of GSK219 (Figure 4, E and F),although the *Trpv4^–/–^* and *Cx3cr1^CreER/+^*:*Trpv4^fl/fl^* mice exhibited no changes in sensorimotor functions (Supplemental Figure 5, A–D). For a positive control, we used resiniferatoxin (RTX) to chemically ablate TRPV1^+^ primary sensory nerves (28) and observed significantly alleviated mechanical allodynia and gait abnormality in mice on day 7 after SNI (Supplemental Figure 6), further confirming the involvement of the nociceptors in the generation of gait abnormality in the SNI mice. Taken together, these findings indicate that microglia-expressed TRPV4 in the spinal cord may be a critical contributor to the generation of neuropathic pain.

### SNI upregulated TRPV4 expression in the spinal cord resident microglia.

**Figure 5:**
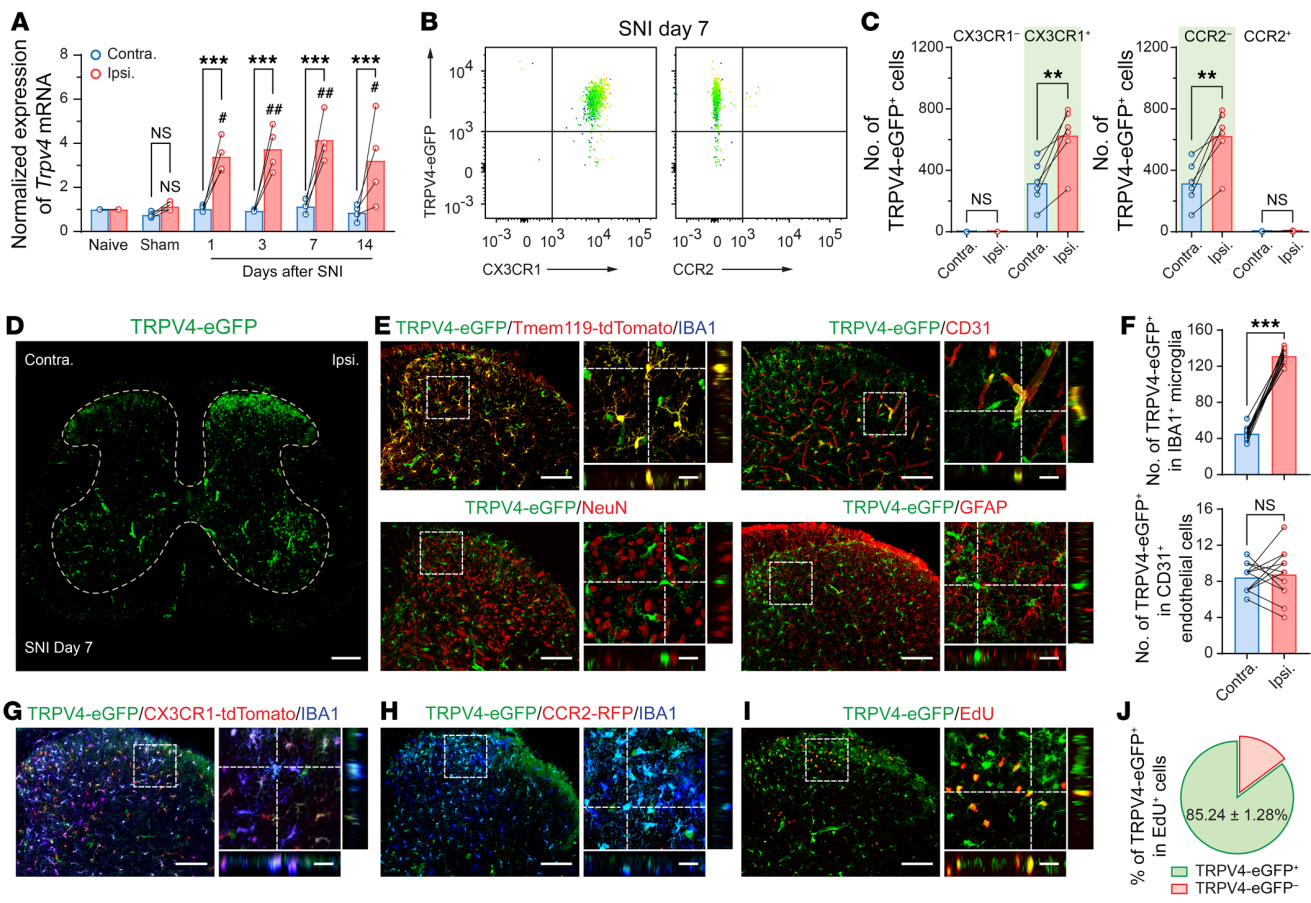
TRPV4 is upregulated in spinal resident microglia following SNI. (**A**) Time course of changes in *Trpv4* mRNA expression in the ipsilateral and contralateral sides of the spinal cord following sham or SNI surgery. *n* = 4 mice per group. ***P < 0.001 by 2-way ANOVA with Bonferroni’s post hoc test. ^#^*P* < 0.05, ^##^*P* < 0.01 versus naive group by 1-way ANOVA with Bonferroni’s post hoc test. (**B** and **C**) Representative flow cytometry plots and count of CX3CR1^+^/TRPV4-eGFP^+^ cells and CCR2^+^/TRPV4-eGFP^+^ cells from the ipsilateral and contralateral sides of the spinal cord of *Trpv4^eGFP^* mice on day 7 after SNI. *n* = 6 mice per group. **P < 0.01 by paired 2-tailed Student’s *t* test. (**D**) Representative image of TRPV4-eGFP expression in the spinal cord of mice on day 7 after SNI. Scale bar: 200 μm. (**E** and **F**) Representative images of TRPV4-expressing cells (TRPV4-eGFP^+^) with IBA1, CD31, NeuN, and GFAP in the spinal dorsal horn of mice on day 7 after SNI (**E**). Quantification of the number of TRPV4-eGFP^+^/IBA1^+^ microglia and TRPV4-eGFP^+^/CD31^+^ endothelial cells. *n* = 12 spinal slices from 3 mice per group. ***P < 0.001 by paired 2-tailed Student’s *t* test (**F**). (**G**) Representative images of TRPV4-eGFP, CX3CR1-tdTomato, and IBA1 coexpression in the spinal dorsal horn of *Trpv4^eGFP^*:*Cx3cr1^CreER/+^*:tdTomato mice on day 7 after SNI. (**H**) Representative images of TRPV4-eGFP, CCR2-RFP, and IBA1 coexpression in the spinal dorsal horn of *Trpv4^eGFP^*:*Ccr2^RFP/+^* mice on day 7 after SNI. (**I** and **J**) Representative images of TRPV4-eGFP and EdU coexpression in the spinal dorsal horn of *Trpv4^eGFP^* mice on day 7 after SNI (**I**) and quantification of the proportion of TRPV4-eGFP^+^/EdU^+^ cells (**J**). *n* = 12 spinal slices from 3 mice per group. Scale bars: 100 μm and 20 μm (zoom). Data are presented as mean ± SEM.

Next, we investigated how SNI affects TRPV4 expression in the spinal cord. Compared with naive or sham mice, *Trpv4* mRNA transcripts were substantially upregulated in the ipsilateral side of the spinal dorsal horn starting within 1 day and lasting for at least 14 days after SNI (Figure 5A). RNAscope assay further revealed the presence of *Trpv4* mRNA transcripts in CX3CR1^+^ microglia in the spinal cord of *Cx3cr1^CreER/+^*:tdTomato mice on day 7 after SNI (Supplemental Figure 7). Moreover, flow cytometric analysis using *Trpv4^eGFP^* mice with CD11b^+^CD45^+^ gating strategy revealed that,compared with the contralateral side, the number of TRPV4-eGFP^+^CX3CR1^+^ — but not TRPV4-eGFP^+^CCR2^+^ — cells was markedly increased in the ipsilateral side of the spinal cord on day 7 after SNI (Figure 5, B and C). In line with the flow cytometry result, immunofluorescence revealed a marked increase in the number of TRPV4-eGFP^+^ cells coexpressing IBA1 or CD31 in the ipsilateral side of the spinal cord on day 7 after SNI (Figure 5, D and E). Of note, TRPV4-eGFP signals were only increased in IBA1^+^ microglia, but not in CD31^+^ endothelial cells (Figure 5F). We also crossed the *Trpv4^eGFP^* mice with the *Cx3cr1^CreER/+^*:tdTomato (Figure 5G) or *Tmem119^CreER/+^*:tdTomato mice (Supplemental Figure 8, A and B) and confirmed that all TRPV4-eGFP signals were expressed by tdTomato^+^/IBA1^+^ resident microglia after SNI.

Interestingly, none of the TRPV4-eGFP^+^ cells expressed CCR2 (Figure 5, B and C), suggesting that TRPV4-eGFP^+^ cells are a subpopulation of spinal resident microglia but not infiltrated blood-borne monocytes. Given that the contribution of infiltrating monocytes to nerve injury–induced microgliosis is still an area of controversy, we further verified whether there was an infiltration of TRPV4-expressing blood-borne monocytes in the spinal dorsal horn in response to SNI. We first crossed a double-transgenic *Trpv4^eGFP^*:*Ccr2^RFP/+^* line and *Trpv4^eGFP^*:*Ccr2^CreER/+^*:tdTomato line bearing eGFP-labeled TRPV4^+^ cells and RFP/tdTomato-labeled CCR2^+^ monocytes and observed no infiltrated CCR2^+^/IBA1^+^ monocytes or CCR2^+^/TRPV4^+^ cells in the spinal dorsal horn on day 7 after SNI (Figure 5H and Supplemental Figure 8C), which is consistent with our flow cytometry observation. Additionally, to exclude the possibility of infiltrating CX3CR1^+^ monocytes in the spinal cord in response to peripheral nerve injury, we treated the *Cx3cr1^CreER/+^*:tdTomato reporter mice with tamoxifen to label all CX3CR1^+^ myeloid cell populations, including spinal resident microglia and blood-borne monocytes. As the cell turnover rates differ between resident microglia and blood-borne monocytes (29), SNI surgery was performed 4 weeks after tamoxifen treatment to allow for repopulation of blood-borne but not resident tdTomato-labeled cells. In this context, tdTomato^–^/IBA1^+^ cells represent infiltrating monocytes, whereas tdTomato^+^/IBA1^+^ cells represent resident microglia which have a much slower turnover rate (30) (Figure 3E). As predicted, we did not detect tdTomato^–^/IBA1^+^ monocytes in the dorsal horn on day 7 after SNI (Supplemental Figure 8D).

Next, we performed i.p. injections of 5-ethynyl-20-deoxyuridine (EdU) to the *Trpv4^eGFP^* mice following SNI surgery. As expected, most of the EdU^+^ microglia in the ipsilateral side of the spinal dorsal horn on day 7 after SNI were eGFP^+^ (Figure 5, I and J). Collectively, these findings indicate that the increased number of the TRPV4-eGFP^+^ cells in the ipsilateral side of the dorsal horn following SNI was due to the proliferation of resident microglia rather than the infiltration of blood-borne monocytes.

### TRPV4 was critically involved in SNI-induced microglial activation and proliferation.

Functional expression of TRPV4 in self-renewal resident microglia provoked the hypothesis that TRPV4 may mediate microglial activation and proliferation in the setting of neuropathic pain. Mediators of inflammation, including tumor necrosis factor-α (TNF-α), interleukins (ILs), and cathepsin S (CatS) are primarily released by microglia and serve as hallmarks of microglial activation (7, 8, 31). As predicted, mRNA transcripts for these inflammatory cytokines were significantly increased in mice subjected to SNI when compared with sham controls, and this SNI-induced upregulation of microglia-derived cytokines was markedly attenuated by *Trpv4* deficiency (Supplemental Figure 9, A–E). On the other hand, the expression levels of IL-18 and BDNF were not changed, although both have been reported to participate in chronic pain processing (7, 8) (Supplemental Figure 9, F and G). These results suggest that TRPV4 function is critically required for SNI-induced spinal microglial activation.

**Figure 6:**
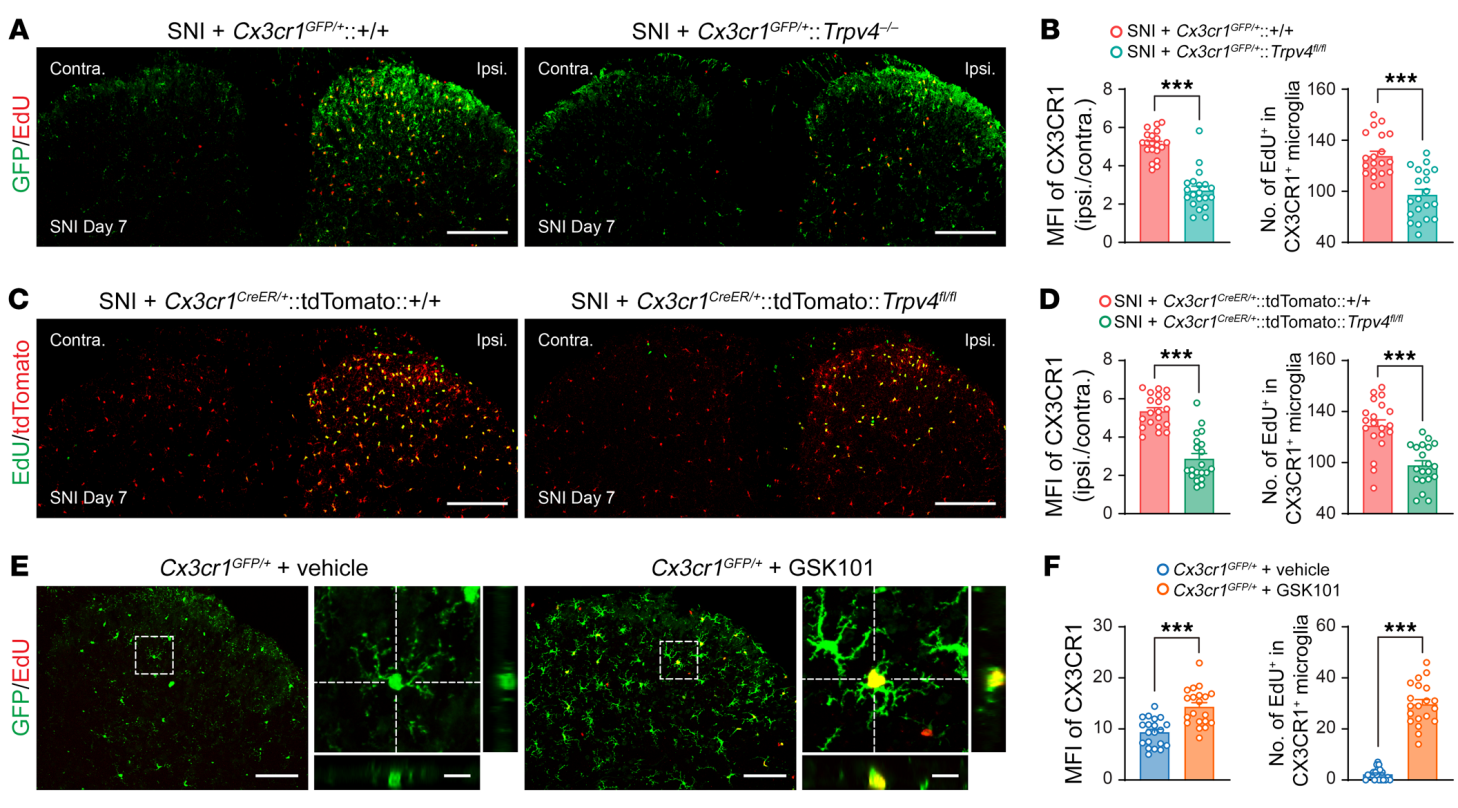
TRPV4 is necessary and sufficient for nerve injury–induced microgliosis following SNI. (**A** and **B**) Representative images of CX3CR1-GFP and EdU coexpression in spinal dorsal horn (**A**) and quantification of MFI of CX3CR1-GFP and number of EdU^+^/CX3CR1-GFP^+^ microglia from *Cx3cr1^GFP/+^* and *Cx3cr1^GFP/+^*:*Trpv4^–/–^* mice on day 7 after SNI (**B**). (**C** and **D**) Representative images of CX3CR1-tdTomato and EdU coexpression in spinal dorsal horn (**C**) and quantification of MFI of CX3CR1-tdTomato and number of EdU^+^/CX3CR1-tdTomato^+^ microglia from *Cx3cr1^CreER/+^*:tdTomato:+/+ and *Cx3cr1^CreER/+^*:tdTomato:*Trpv4^fl/fl^* mice on day 7 after SNI (**D**). EdU was i.p. administrated once per day for 3 consecutive days starting on day 1 after SNI. *n* = 20 spinal slices from 4 mice per group. ***P < 0.001 by unpaired 2-tailed Student’s *t* test. Scale bar: 200 μm. (**E** and **F**) Representative images of CX3CR1-tdTomato and EdU coexpression in spinal dorsal horn (**E**) and quantification of the MFI of CX3CR1-tdTomato and number of EdU^+^/CX3CR1-tdTomato^+^ microglia from *Cx3cr1^GFP/+^* mice following GSK101 administration (**F**). Coadministration of i.t. GSK101 (1 μg) and i.p. EdU injection was performed once per day for 3 consecutive days. *n* = 20 spinal slices from 4 mice per group. ***P < 0.001 by unpaired 2-tailed Student’s *t* test. Scale bars: 100 μm and 20 μm (zoom). Data are presented as mean ± SEM.

To further investigate if TRPV4 contributes to SNI-induced microglial activation and proliferation, we performed i.p. injections of EdU for 3 consecutive days starting on day 1 after SNI. Strikingly, a substantial increase in CX3CR1, whether GFP or tdTomato, fluorescence intensity and the number of EdU^+^/CX3CR1^+^ cells was detected in the ipsilateral dorsal horn of both *Cx3cr1^GFP/+^* mice and *Cx3cr1^CreER/+^*:tdTomato mice on day 7 after SNI. However, this SNI-induced microglial proliferation was significantly reduced in both *Cx3cr1^GFP/+^*:*Trpv4^–/–^* mice and *Cx3cr1^CreER/+^*:tdTomato:*Trpv4^fl/fl^* mice (Figure 6, A–D), as well as *Tmem119^CreER/+^*:tdTomato mice treated with GSK219 (Supplemental Figure 8, E and F). Similarly, the SNI-induced increase in fluorescence intensity of IBA1 expression and the number of IBA1^+^ microglia were also reduced in both global *Trpv4* KO (Supplemental Figure 9, H and I) and microglia-specific *Trpv4* cKO mice (Supplemental Figure 9, J and K). Conversely, we further tested if the chemical activation of TRPV4 was sufficient to promote microglial activation and proliferation. Indeed, i.t. injection of GSK101 combined with i.p. injection of EdU in *Cx3cr1^GFP/+^* mice for 3 consecutive days caused a noticeable increase in CX3CR1-GFP fluorescence intensity and the number of EdU^+^/CX3CR1^+^ cells in the spinal dorsal horn (Figure 6, E and F), suggesting that activation of TRPV4 was sufficient to produce microglial activation and proliferation.

### Spinal activation of TRPV4 was sufficient to promote excitatory neuronal hyperactivity and produce acute mechanical pain.

**Figure 7:**
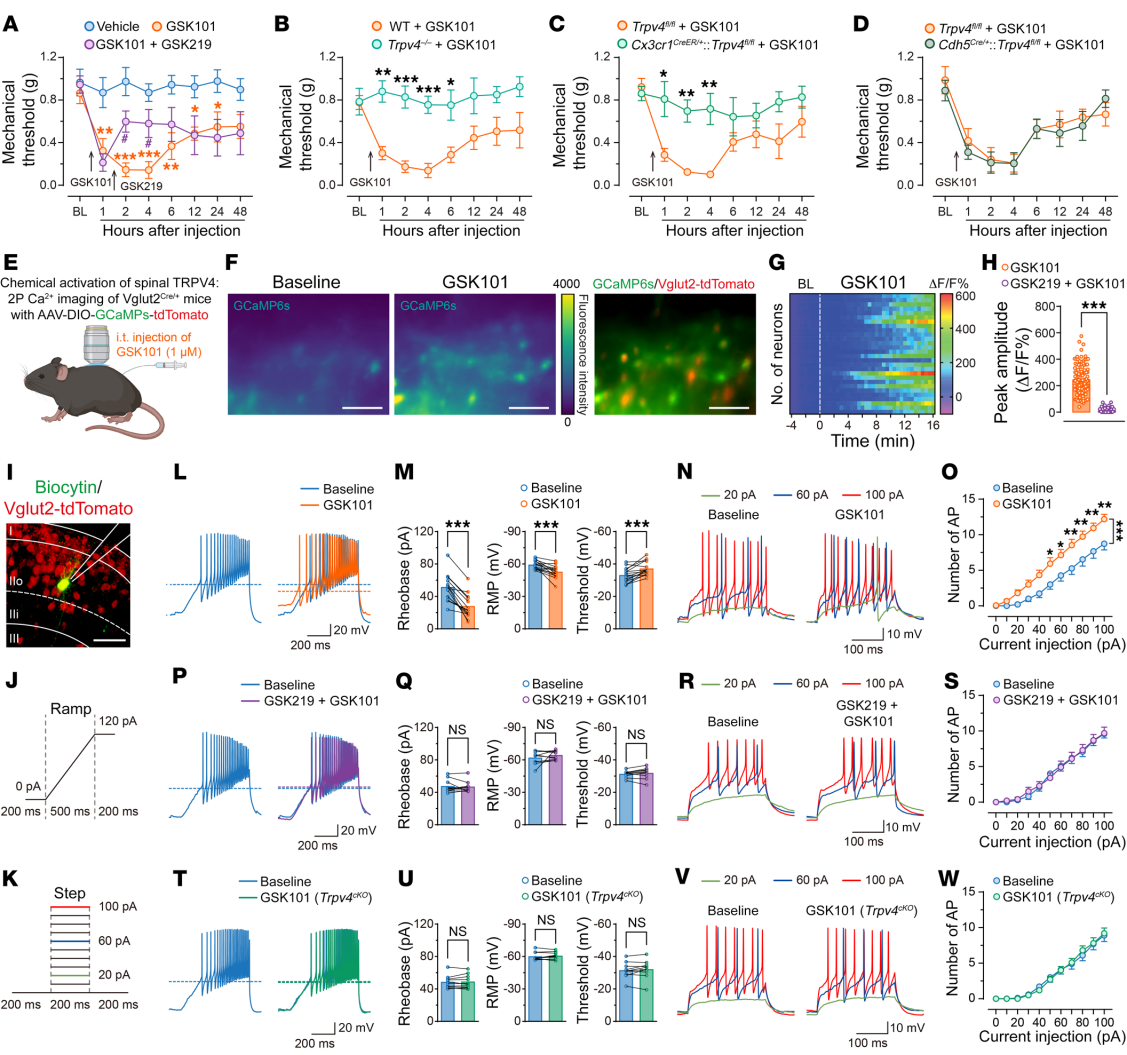
Activation of TRPV4 by GSK101 induces pain hypersensitivity and hyperactivity of spinal excitatory neurons. (**A**– **D**) Time course of paw withdrawal threshold following i.t. injection of GSK101 in vehicle-treated and GSK219-treated WT mice (**A**), WT control littermates and *Trpv4^–/–^* mice (**B**), *Trpv4^fl/fl^* control littermates and *Cx3cr1^CreER/+^*:*Trpv4^fl/fl^* mice (**C**); *Trpv4^fl/fl^* control littermates and *Cdh5^Cre/+^*:*Trpv4^fl/fl^* mice (**D**). GSK219 (25 μg) was i.t. injected 1.5 hours after GSK101 (1 μM) administration. *n* = 7–10 mice per group. ^#^*P* < 0.05 versus vehicle group; *P < 0.05, **P < 0.01, ***P < 0.001 versus GSK101 group by 2-way repeated ANOVA with Bonferroni’s post hoc test. (**E**–**H**) Schematic illustration of in vivo 2-photon Ca^2+^ imaging (**E**), representative 2-photon images (**F**), Δ*F*/*F* hot map (**G**), and Δ*F*/*F* peak amplitude (**H**) of spinal Vglut2^+^ neurons responses to GSK101 (1 μM) and GSK219 (25 μg) i.t. injection. *n* = 105–120 neurons from 4 mice. ***P < 0.001 by unpaired 2-tailed Student’s *t* test. Scale bar: 50 μm. (**I**–**K**) Representative recorded Vglut2-tdTomato^+^ neuron in the lamina IIo of spinal cord slice (**I**), ramp protocol of depolarizing current was applied to assess the rheobase, RMP, and threshold (**J**), and step protocol of depolarizing currents was applied to assess the number of AP firings (**K**). (**L**–**S**) Representative traces and quantification of AP firings from *Vglut2^Cre/+^*:tdTomato mice with GSK101 (300 nM) and GSK219 (300 nM) perfusion. *n* = 10–15 neurons from 3–4 mice. (**T**–**W**) Representative traces and quantification of AP firings from *Cx3cr1^CreER/+^*:*Trpv4^fl/fl^* mice with GSK101 perfusion. *n* = 10 neurons from 3 mice. ***P < 0.001 by paired 2-tailed Student’s *t* test (**M**, **Q**, and **U**), *P < 0.05, **P < 0.01, ***P < 0.001 by 2-way repeated ANOVA with Bonferroni’s post hoc test (**O**, **S**, and **W**). Data are presented as mean ± SEM.

Bidirectional microglia-neuron communications are critical to the generation and maintenance of neuropathic pain (7, 8). We reasoned that if TRPV4 was a critical mediator of SNI-induced mechanical allodynia and microglial activation, direct chemical activation of spinal TRPV4 alone should be sufficient to cause pain hypersensitivity. Indeed, a single i.t. injection of GSK101 markedly reduced the paw-withdrawal threshold, which was reversed by coapplied GSK219 (Figure 7A). Moreover, GSK101-induced mechanical allodynia was significantly abolished in global *Trpv4* KO mice (Figure 7B) and microglia-specific *Trpv4* cKO mice (Figure 7C), but not in endothelial cell-specific or neuron-specific *Trpv4* cKO mice (Figure 7D and Supplemental Figure 4I). Interestingly, the mechanical hypersensitivity induced by i.t. application of GSK101 was reversible between 24–48 hours after injection, suggesting an acute and reversible activation/sensitization of pain-transmitting neurons in the spinal cord.

We further examined the activity of the spinal excitatory neurons following GSK101 administration by monitoring [Ca^2+^]_i_ levels in spinal Vgult2^+^ neurons from naive *Vgult2^Cre/+^* mice using virus-mediated expression of the Cre-inducible Ca^2+^ indicator GCaMP6s (AAV-DIO-GCaMP6s-tdTomato). Using in vivo 2-photon imaging, we found that i.t. injections of GSK101 evoked a rise in [Ca^2+^]_i_ transients in the spinal Vgult2^+^ interneurons, which was abolished by GSK219 pretreatment (Figure 7, E–H). Moreover, using ex vivo patch-clamp recording in lamina IIo Vgult2^+^ interneurons from naive *Vgult2^Cre/+^*:tdTomato mice with ramp and step protocols of depolarizing current application (Figure 7, I–K), we also observed that acute perfusion with GSK101 significantly decreased the rheobase and increased the resting membrane potential (RMP), action potential (AP) threshold, and frequency compared with baseline (15/24 neurons from 4 mice; Figure 7, L–O), which was also abolished by GSK219 pretreatment (10/10 neurons from 3 mice; Figure 7, P–S). Importantly, no GSK101-induced increases in AP firings were detected in lamina IIo interneurons from microglia-specific *Trpv4* cKO mice (10/10 neurons from 3 mice, Figure 7, T–W). These findings indicate that activation of spinal microglia-expressed TRPV4 was sufficient to produce excitatory neuronal hyperactivity and drive acute mechanical hypersensitivity.

Next, we asked if TRPV4 activation also affected disinhibition from inhibitory interneurons, which is also a major feature of neuropathic pain (32, 33). Surprisingly, GSK101 did not significantly affect the rheobase, RMP, AP threshold, and frequency, compared with baseline by using ex vivo patch-clamp recording in lamina II Vgat^+^ inhibitory interneurons from naive *Vgat^Cre/+^*:tdTomato mice with ramp and step protocols of depolarizing current application (Supplemental Figure 10, A–K). More importantly, GSK219 had no effect on AP firings in the inhibitory interneurons from *Vgat^Cre/+^*:tdTomato mice on day 7 after SNI (Supplemental Figure 10, L–O), suggesting that TRPV4 activation in spinal microglia selectively contributed to gain in excitability in the spinal excitatory neurons, but was not relevant to disinhibition in the spinal inhibitory neurons.

### Microglia-expressed TRPV4 was necessary for SNI-induced functional and structural plasticity of spinal excitatory neurons.

**Figure 8:**
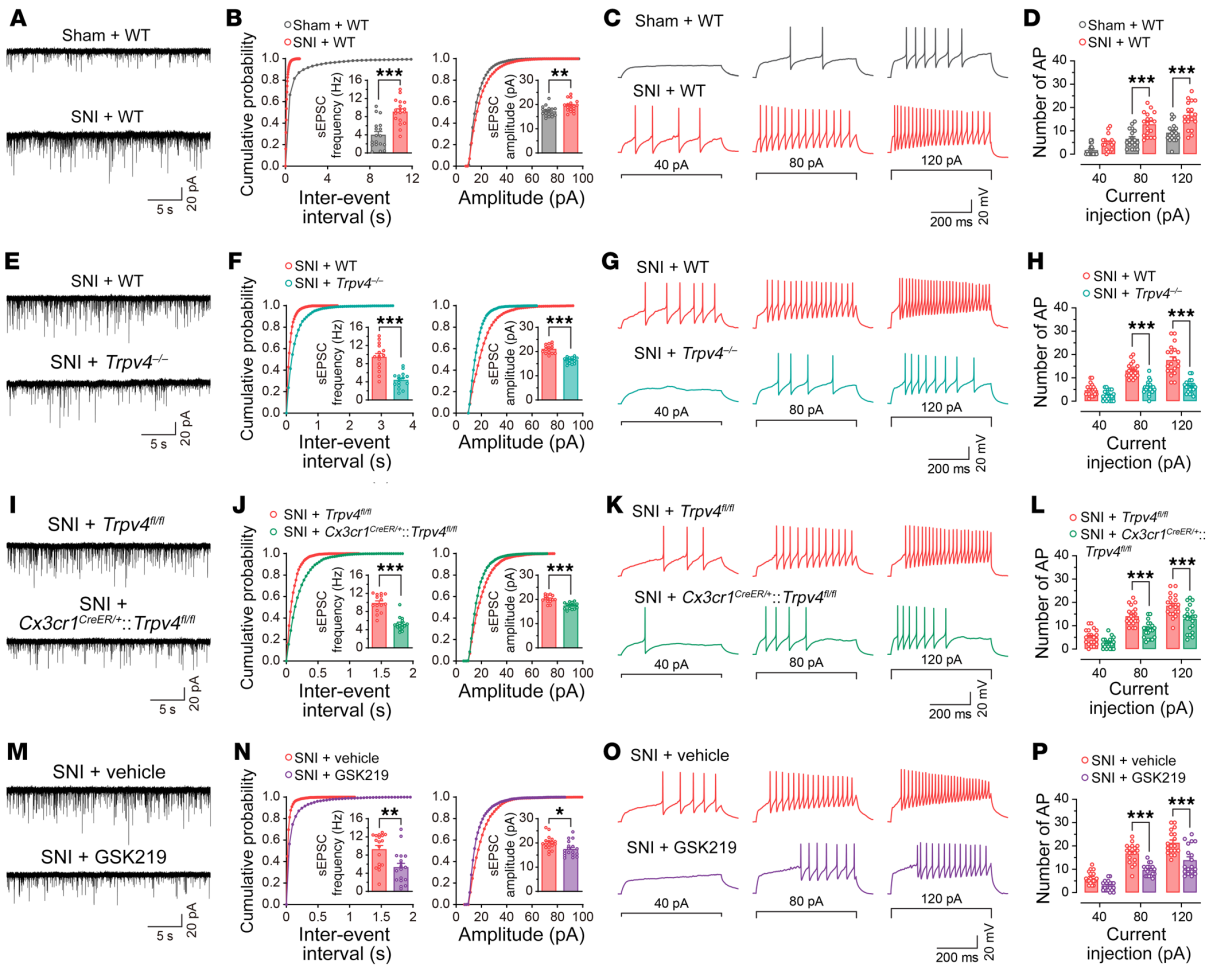
Genetic or pharmacological inhibition of TRPV4 reduces SNI-induced neuronal hyperactivity and inhibits dendritic spine remodeling in spinal lamina IIo neurons. (**A**–**D**) Representative traces and quantification of the frequency and amplitude of sEPSCs (**A** and **B**), and the number of AP firings (**C** and **D**) in spinal lamina IIo neurons from WT mice on day 7 after sham or SNI. *n* = 18 neurons from 4 mice per group. (**E**–**H**) Representative traces and quantification of the frequency and amplitude of sEPSCs (**E** and **F**), and the number of AP firings (**G** and **H**) in spinal lamina IIo neurons from WT control littermate and *Trpv4^–/–^* mice on day 7 after SNI. *n* = 15–20 neurons from 4 mice per group. (**I**–**L**) Representative traces and quantification of the frequency and amplitude of sEPSCs (**I** and **J**), and the number of AP firings (**K** and **L**) in spinal lamina IIo neurons from *Trpv4^fl/fl^* control littermates and *Cx3cr1^CreER/+^*:*Trpv4^fl/fl^* mice on day 7 after SNI. *n* = 15–20 neurons from 4 mice per group. (**M**–**P**) Representative traces and quantification of the frequency and amplitude of sEPSCs (**M** and **N**), and the number of current step-induced AP firings (**O** and **P**) in spinal lamina IIo neurons from WT mice treated with vehicle or GSK219 (30 mg/kg, i.p., once per day from days 1– 7 after SNI) on day 7 after SNI. *n* = 20 neurons from 4 mice per group. *P < 0.05, **P < 0.01, ***P < 0.001 by unpaired Student’s t-test (**B**, **F**, **J**, and **N**), ***P < 0.001 by 2-way repeated ANOVA with Bonferroni’s post hoc test (**D**, **H**, **L**, and **P**). Data are represented as mean ± SEM.

It is well established that functional and structural plasticity at various sites in somatosensory pain circuits is closely related to the transition from acute to chronic pain (34). To test whether blocking microglia-expressed TRPV4 was sufficient to suppress spinal synaptic plasticity in the setting of neuropathic pain, we first measured spinal excitatory synaptic transmission and membrane excitability by detecting spontaneous excitatory postsynaptic currents (sEPSCs) and AP firings in lamina IIo interneurons. As predicted, mice subjected to SNI exhibited increased frequency and amplitude of sEPSCs, as well as AP firing frequency, compared with sham control mice at day 7 after SNI (Figure 8, A–D). Consistent with the behavioral phenotypes, global *Trpv4* KO (Figure 8, E–H) and microglia-specific *Trpv4* cKO (Figure 8, I–L) mice, as well as mice treated with GSK219 (Figure 8, M–P) exhibited significantly reduced frequency and amplitude of sEPSCs, as well as AP firing frequency following SNI, when compared with their respective controls. Moreover, acute perfusion with GSK219 instantaneously diminished the frequency and amplitude of sEPSCs, as well as AP firing frequency in Vgult2^+^ neurons on day 7 after SNI (Supplemental Figure 11). Together, our results demonstrate that microglia-expressed TRPV4 was required to mediate excitatory synaptic transmission and promote excitability in the spinal cord following peripheral nerve injury.

Given that central sensitization also depends on the structural plasticity of spinal neurons in chronic pain states (34), we sought to investigate whether peripheral nerve injury induced dendrite spine remodeling beyond functional plasticity in the spinal neurons and whether it relies on the function of microglia-expressed TRPV4. Indeed, neurons in spinal lamina IIo stained by microinjection of biocytin exhibited a significant increase in the density of dendrite spines on day 7 after SNI compared with neurons from the sham control mice (Supplemental Figure 12, A and B). This SNI-induced increase in dendrite spine density was reduced in both global *Trpv4* KO (Supplemental Figure 12, C and D) and microglia-specific *Trpv4* cKO (Supplemental Figure 12, E and F) mice, as well as mice treated with repeated i.p. injections of GSK219 for 7 days after SNI (Supplemental Figure 12, G and H) when compared with their respective controls. These findings demonstrate that the transition of synaptic potentiation to structural alteration in the spinal cord pain circuit was dependent on the function of microglia-expressed TRPV4, which may underlie pain chronicity.

### Microglia-expressed TRPV4 mediated functional and structural plasticity of spinal excitatory neurons through LCN2.

**Figure 9:**
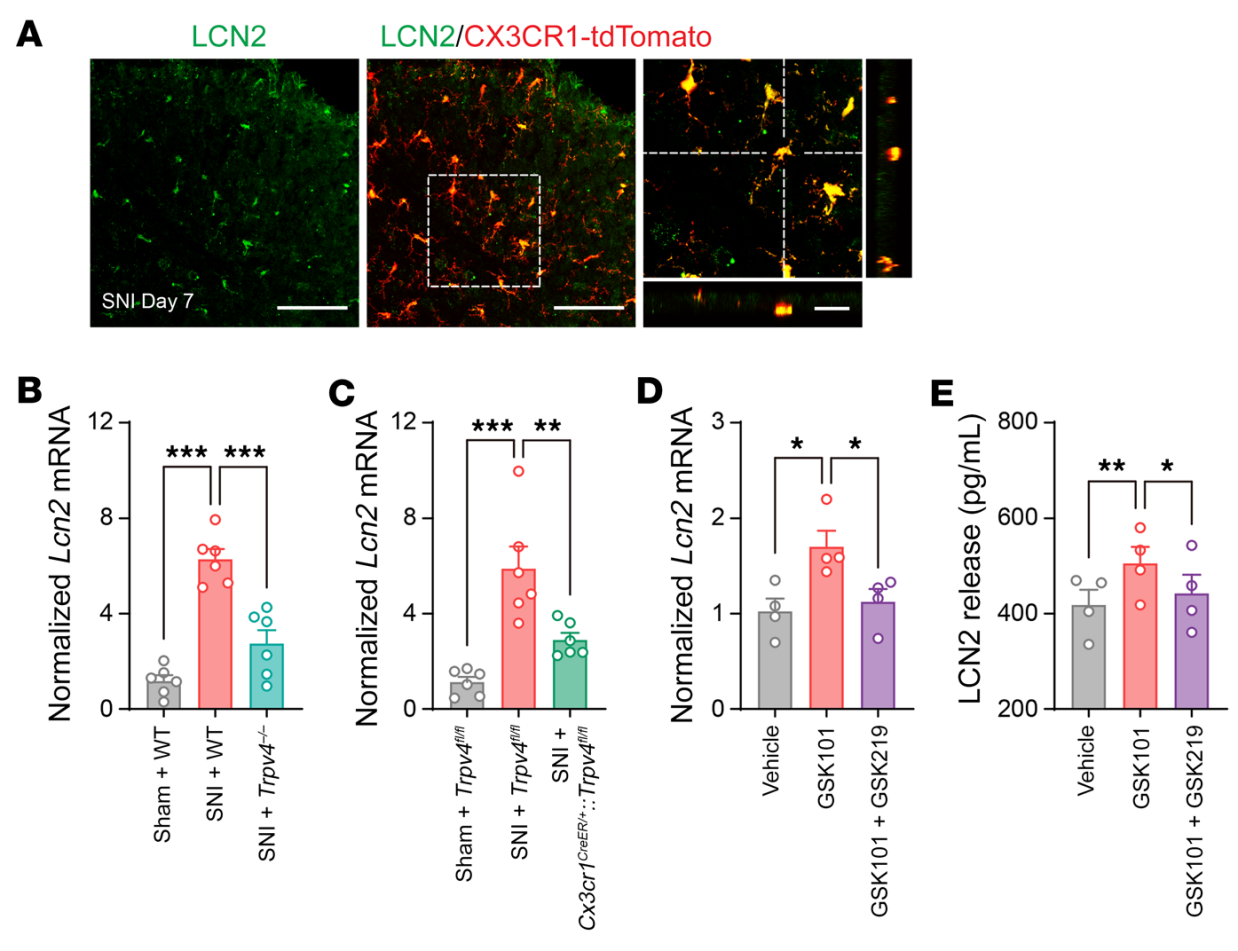
TRPV4 is necessary and sufficient for LCN2 expression in the spinal microglia. (**A**) Representative images of LCN2 antibody with CX3CR1-tdTomato in the spinal dorsal horn of *Cx3cr1^CreER/+^*:tdTomato mice on day 7 after SNI. Scale bars: 100 μm and 20 μm (zoom). (**B** and **C**) Relative expression levels of *Lcn2* mRNA in the ipsilateral sides of the spinal cord from WT mice and *Trpv4^–/–^* mice (**B**), *Trpv4^fl/fl^* control littermates and *Cx3cr1^CreER/+^*:*Trpv4^fl/fl^* mice (**C**) on day 7 after sham or SNI surgery. *n* = 6 mice per group. Statistics were determined by 1-way ANOVA with Bonferroni’s post hoc test. (**D** and **E**) Relative expression levels of *Lcn2* mRNA in the cultured primary spinal microglia (**D**) and LCN2 release in the culture supernates (**E**) with GSK101 (300 nM) and GSK219 (30 nM) treatment. *n* = 4 wells per group from 2 independent experiments. Statistics were determined by 1-way ANOVA with Bonferroni’s post hoc test. Data are presented as mean ± SEM. *P < 0.05, **P < 0.01, ***P < 0.001.

**Figure 10:**
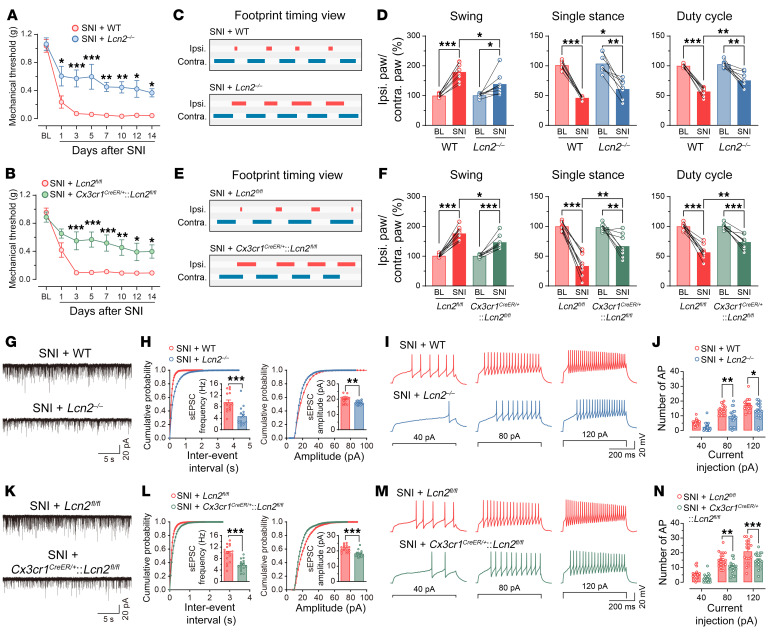
Genetic inhibition of LCN2 in the spinal microglia reduces SNI-induced neuronal hyperactivity in spinal lamina IIo neurons. (**A** and **B**) Time course of paw withdrawal threshold following SNI in WT control littermates and *Lcn2^–/–^* mice (**A**), *Lcn2^fl/fl^* control littermates and *Cx3cr1^CreER/+^*:*Lcn2^fl/fl^* mice (**B**). *n* = 7–10 mice per group. Statistics were determined by 2-way repeated ANOVA with Bonferroni’s post hoc test. (**C**–**F**) Representative footprint timing view and CatWalk gait analysis of WT control littermate and *Lcn2^–/–^* mice (**C** and **D**), *Lcn2^fl/fl^* control littermates and *Cx3cr1^CreER/+^*:*Lcn2^fl/fl^* mice (**E** and **F**) on day 7 after SNI. *n* = 8–10 mice per group. Statistics were determined by paired or unpaired 2-tailed Student’s *t* test. (**G**–**J**) Representative traces and quantification of the frequency and amplitude of sEPSCs (**G** and **H**), and the number of AP firings (**I** and **J**) in spinal lamina IIo neurons from WT control littermate and *Lcn2^–/–^* mice on day 7 after SNI. *n* = 16–20 neurons from 4 mice per group. (**K**–**N**) Representative traces and quantification of the frequency and amplitude of sEPSCs (**K** and **L**), and the number of AP firings (**M** and **N**) in spinal lamina IIo neurons from *Lcn2^fl/fl^* control littermates and *Cx3cr1^CreER/+^*:*Lcn2^fl/fl^* mice on day 7 after SNI. *n* = 16–20 neurons from 4 mice per group. **P < 0.01, ***P < 0.001 by unpaired 2-tailed Student’s *t* test (**H** and **L**), Statistics were determined by 2-way repeated ANOVA with Bonferroni’s post hoc test (**J** and **N**). Data are presented as mean ± SEM. *P < 0.05, **P < 0.01, ***P < 0.001.

Microglia communicate with other cell types in the CNS by releasing soluble factors (7, 35). We showed that many proinflammatory cytokines can be released from activated microglia after SNI (Supplemental Figure 9, A–E). Notably, published studies demonstrated that immune cell-derived proinflammatory cytokines generally either modulate or have no effect on dendritic spine density in the CNS (36–38). Interestingly, we found that TRPV4 activation in microglia also markedly upregulated the production of LCN2, a secreted protein that was shown to be an important regulator of neuronal excitability in the CNS (31, 39, 40). Further, LCN2 expression is significantly upregulated in a mouse model of chronic neuropathic pain caused by spinal nerve ligation (30). Importantly, LCN2 expression and function are closely related to spine density and membrane excitability in the CNS in a region-specific manner (39, 41), prompting us to hypothesize that LCN2 may be involved in TRPV4-mediated spinal synaptic plasticity and neuropathic pain. To test this hypothesis, we first checked LCN2 expression in the spinal cord and determined the role of TRPV4 in LCN2 production. Following SNI, LCN2 was specifically expressed in the spinal dorsal horn CX3CR1^+^ microglia (Figure 9A) but was absent from the global *Lcn2* KO (*Lcn2^–/–^*) and microglia-specific *Lcn2* cKO (*Cx3cr1^CreER/+^*:*Lcn2^fl/fl^*) mice (Supplemental Figure 13, A and B). Strikingly, *Lcn2* mRNA expression was markedly increased in the spinal dorsal horn following SNI when compared with the sham control group, and this upregulation was substantially reduced in both global *Trpv4* KO (Figure 9B) and microglia-specific *Trpv4* cKO (Figure 9C) mice. Consistent with the genetic ablation studies, chemical activation of TRPV4 by GSK101 for 48 hours induced *Lcn2* mRNA upregulation in cultured spinal microglia (Figure 9D) and promoted the release of LCN2 protein in cell culture supernates (Figure 9E), which were suppressed by coapplication of GSK219. Moreover, both global *Lcn2* KO and microglia-specific *Lcn2* cKO mice exhibited improved mechanical allodynia and gait abnormality following SNI (Figure 10, A–F) without obvious changes in sensorimotor behaviors (Supplemental Figure 13, C–F), suggesting that spinal LCN2 was required to generate the SNI-induced mechanical pain hypersensitivity in a TRPV4-dependent manner.

While LCN2 has been implicated in driving both inflammatory and neuropathic pain, its role in spinal synaptic plasticity remains unknown. Consistent with in vivo pain behavioral studies, we found that both global and microglia-specific ablation of *Lcn2* reduced the SNI-induced frequency and amplitude of sEPSCs, AP firing frequency (Figure 10, G–N), and the density of dendrite spines (Supplemental Figure 12, I–L) on day 7 after SNI. Conversely, i.t. injection of recombinant LCN2 for 3 consecutive days was sufficient to increase the frequency and amplitude of sEPSCs, AP firing frequency (Supplemental Figure 13, G–J), and the density of dendrite spines (Supplemental Figure 13, O and P). In addition, *Lcn2* deficiency effectively reduced the increase in frequency and amplitude of sEPSCs, AP firing frequency (Supplemental Figure 13, K–N), and the density of dendrite spines (Supplemental Figure 13, Q and R) induced by i.t. injection of GSK101 for 3 consecutive days. Collectively, these results demonstrate that LCN2 was a key downstream mediator of microglial TRPV4 signaling that promotes spinal synaptic plasticity and neuropathic pain.

## Discussion

Nociceptive TRP channels expressed by primary nociceptors are key mediators of both inflammatory and neuropathic pain, which is well supported by both genetic and pharmacological studies (42). Moreover, the expression and function of nociceptive TRP channels are often regulated by tissue inflammation and nerve injury, and serve as promising drug targets for the treatment of chronic pain (43–46). However, the role of centrally expressed TRP channels in neuropathic pain is under-studied. Here, we provide several lines of evidence for a critical role of TRPV4 channels expressed by spinal microglia in the neuroimmune axis leading to neuropathic pain. First, by using the SNI-induced neuropathic pain model, we showed that both global and microglia-specific ablation of TRPV4 function significantly attenuated mechanical pain in both male and female mice. In addition, either systematic or spinal administration of a potent TRPV4-specific antagonist GSK219 prevented the development of mechanical pain, suggesting a potential therapeutic effect of blocking TRPV4 in chronic neuropathic pain. Second, we demonstrated that microglial activation and proliferation induced by SNI required the function of microglia-expressed TRPV4. Third, we provided evidence that SNI-enhanced neuronal excitability and dendritic spine remodeling in lamina IIo spinal neurons were mediated by TRPV4, and TRPV4 promoted spinal neuroplasticity through microglia-derived LCN2. Taken together, we believe that our studies shed new light on the cellular and molecular mechanisms underlying neuropathic pain in the spinal cord and identify TRPV4 as a promising molecular target for chronic neuropathic pain treatment.

Using *Trpv4^eGFP^* reporter mice, we identified selective TRPV4 expression in IBA1^+^ spinal microglia and CD31^+^ endothelial cells, but did not find TRPV4 expression in TRPV1^+^ central afferent terminals (47). This was surprising since previous studies have shown that TRPV4 is expressed in primary afferent neurons and is involved in pain processing, although Ca^2+^ imaging using cultured DRG neurons also revealed the absence of a GSK101-induced response (48, 49). Following SNI surgery, TRPV4 expression was time-dependently increased in the spinal dorsal horn microglia, but not in NeuN^+^ neurons, GFAP^+^ astrocytes, or CD31^+^ endothelial cells. Moreover, we showed that SNI-induced mechanical pain was alleviated in microglia-specific *Trpv4* cKO mice in both sexes, but not affected in the nociceptor-specific, endothelial cell-specific, or monocyte-specific *Trpv4* cKO mice. These results disclose an immunogenic action of TRPV4 in SNI-induced neuropathic pain. Interestingly, our results suggest that the contribution of TRPV4 to mechanical hypersensitivity does not appear to be sexually dimorphic, which is of interest in light of the previous report that spinal microglia in male mice, but not in female mice, reduced nerve injury-induced neuropathic pain (50). Future studies are required to understand the precise role of sex in TRPV4-dependent neuropathic pain. In addition to measuring reflex pain behavior evoked by mechanical stimulation, we adopted an automated CatWalk system to examine changes in pain-related gait parameters after SNI. We demonstrated that TRPV4 was also required by SNI-induced gait alterations by using both pharmacological and genetic inhibition approaches, pointing a role of TRPV4 in SNI-induced nonreflexive pain behaviors. It should be noted that, although we used mice in which the TRPV1^+^ fibers were ablated to demonstrate a correlation between pain behaviors and gait abnormalities, we cannot completely rule out the role of TRPV4 in the locomotor deficits, considering that these animals also have a major spinal microglial activation that extends to the ventral horns.

TRPV4 upregulation induced by peripheral nerve injury is associated with remarkable microgliosis in the spinal dorsal horn. Although myeloid expansion in the CNS can result from either the infiltration of circulating monocytes or by self-renewal of resident microglia (51, 52), we found no infiltration of blood-borne monocytes into the spinal dorsal horn upon SNI, which is consistent with published studies (30, 53). Instead, we showed that SNI-induced TRPV4-dependent microgliosis was driven primarily by the proliferation of resident microglia, as more than 80% of TRPV4-eGFP^+^ cells were EdU^+^ self-renewal microglia. Moreover, the increase in the number of EdU^+^/CX3CR^+^ and IBA1^+^ microglia in the spinal dorsal horn following SNI was inhibited by either global or microglia-specific ablation of TRPV4 function. Furthermore, *Trpv4* deficiency also markedly reduced the upregulation of microglia-derived proinflammatory cytokines induced by SNI (7, 54). Collectively, these findings demonstrate that peripheral nerve injury-induced TRPV4 expression in the self-renewal resident spinal microglia was strongly associated with SNI-induced microglial activation and proliferation.

Another striking observation was that spinal activation of microglia-expressed TRPV4 was sufficient to produce reversible acute mechanical pain. Using in vivo 2-photon Ca^2+^ imaging and ex vivo patch-clamp recording, we also showed that acute administration of GSK101 could induce Ca^2+^ transients and hyperactivity of spinal Vglut2^+^ excitatory interneurons, which form a nociceptive circuit with input from primary sensory afferents and output to projection neurons (28). We further demonstrated that mice treated with SNI exhibited an increase in sEPSCs and AP firings in the spinal excitatory neurons in a TRPV4-dependent manner. Importantly, acute application of GSK219 rapidly suppressed SNI-induced enhancement of both electrical and synaptic activities, suggesting that TRPV4 channels are likely constitutively active during SNI-induced neuropathy and might serve as a promising drug target for treating chronic neuropathic pain. It is well established that dendritic spine remodeling in the spinal dorsal horn is associated with synaptic structural plasticity and nociceptive hypersensitivity (24, 55). We further confirmed that the SNI-induced increase in dendritic spine density was also dependent on microglia-expressed TRPV4 channel function. Our findings suggest that microglia-expressed TRPV4 plays an essential role in promoting the excitability of excitatory spinal neurons, synaptic potentiation, and structural plasticity, which are critical cellular mechanisms underlying the transition from acute to chronic neuropathic pain in the context of peripheral nerve injury.

Emerging evidence indicates that LCN2 is synthesized and secreted from activated glial cells and is recognized as a modulatory factor for neuronal excitability and synaptic plasticity in response to CNS disorders (56). Recent studies also demonstrate that centrally expressed LCN2 plays a crucial role in the pathogenesis of pain and itch sensitization (31, 40, 57). We show herein that LCN2 was expressed in the spinal microglia and was upregulated after SNI in a TRPV4-dependent manner. Moreover, both global and microglia-specific ablation of *Lcn2* suppressed SNI-induced functional and structural synaptic plasticity in the spinal cord, as well as neuropathic pain-related behaviors, which mirror the phenotypes of *Trpv4* deficiency mice. In addition, LCN2 is sufficient for synaptic plasticity and is necessary for GSK101-induced synaptic plasticity in the spinal cord, suggesting that LCN2 is a critical mediator downstream of TRPV4 signaling in spinal microglia. Interestingly, Jeon et al. reported that the LCN2 receptor 24p3R is expressed by neurons in the spinal cord (31), suggesting that microglia-derived LCN2 likely activates the 24p3R in the spinal neurons and promotes synaptic plasticity through microglia-neuronal crosstalk in the setting of peripheral nerve injury-induced neuropathic pain. It is noteworthy that in hippocampal pyramidal neurons, *Lcn2* deficiency facilitates neuronal activity and dendritic spine density (39), which opposes our findings and findings by others in the spinal dorsal horn (40, 58). Although underlying mechanisms through which LCN2 differentially regulates synaptic alterations in the hippocampus and spinal cord are still unknown, it is conceivable that the function of activated microglia in the CNS might be region-specific (7, 24).

In summary, we believe that we have discovered a central role of spinal resident microglia-expressed TRPV4 in transforming peripheral nerve injury to spinal central sensitization and neuropathic pain. Our study provides evidence that spinal TRPV4 mediates microglial activation and proliferation and promotes synaptic transmission and plasticity of excitatory neurons through release of LCN2. Therefore, we have identified an integrated neuroimmune axis that relies on TRPV4 function in pain chronicity, and TRPV4 emerged as a molecular target for the treatment of chronic neuropathic pain.

## Methods

### Animals.

Adult (8–12 weeks) male and/or female C57BL/6J mice (The Jackson Laboratory), *Trpv4^eGFP^* (The Mutant Mouse Resource and Research Center [MMRRC]), *Trpv4*^–/–^ (donated by Makoto Suzuki and Atsuko Mizuno [Jichi Medical School, Minamikawachi, Tochigi, Japan]), *Trpv4^flox^* (13), *Cx3cr1^CreER^* (The Jackson Laboratory), *Tmem119^CreER^* (The Jackson Laboratory), tdTomato (Ai9, The Jackson Laboratory), GCaMP6s (Ai96, The Jackson Laboratory), *Cx3cr1^GFP^* (The Jackson Laboratory), *Ccr2^RFP^* (The Jackson Laboratory), *Ccr2^CreER^* (provided in-house), *Trpv1^Cre^* (donated by Mark Hoon [National Institute of Dental and Craniofacial Research, Bethesda, Maryland, USA]), *Cdh5^Cre^* (The Jackson Laboratory), *Vglut2^Cre^* (The Jackson Laboratory), *Vgat^Cre^* (The Jackson Laboratory), *Lcn2*^–/–^ (The Jackson Laboratory), and *Lcn2^flox^* (donated by Jack Cowland [Rigshospitalet, Copenhagen, Denmark], Bin Gao [National Institute on Alcohol Abuse and Alcoholism, Bethesda, Maryland, USA], and Grace Guo [Rutgers University, Piscataway, New Jersey, USA]) mice were used for this study. Mice were bred and maintained (3–5 mice per cage) in individually ventilated cages on a 12 hour light/12 hour dark cycle, and with ad libitum access to food and water. The experimental holding room was supplied with HEPA-filtered air and had temperature and humidity control maintained at 22°C and 40%, respectively. All of the experimental procedures were approved by the IACUC of Washington University School of Medicine. All mice were randomly allocated to different experimental groups by the lab managers, all of whom were blinded to the experimental design.

### Spared nerve injury model of neuropathic pain.

The SNI procedure was performed as described previously (25, 59). In brief, mice were anesthetized with 2% isoflurane and the skin and muscle of the right thigh were incised to explore the sciatic nerve, which consists of the sural, common peroneal, and tibial nerves. After exploration, the common peroneal and tibial nerves were ligated using nonabsorbent 5-0 chromic gut sutures, then the nerves were transected; roughly 1 mm sections from the point of transection were removed. The skin was stitched and disinfected with betadine. The sham surgery was the same as the SNI procedure, but the nerves were not cut.

### Drugs and administration.

Intraperitoneal administration of tamoxifen (Sigma-Aldrich) for 5 consecutive days at 100 mg/kg in 10 mg/mL of corn oil (Sigma-Aldrich) was used to for the induction of Cre expression in inducible Cre-driver lines. Animals were used for experiments 1 week, for *Ccr2^CreER^* mice and *Tmem119^CreER^* mice, or 4 weeks, for *Cx3cr1^CreER^* mice, after tamoxifen administration.

Under anesthesia with 2% isoflurane, i.t. injection was performed with a 30-G needle attached to a Hamilton microsyringe inserted between the L5 and L6 vertebrae and then punctured through the dura. The following drugs with corresponding doses were used for i.t. injection experiments: GSK1016790A (GSK101, 1 μM in 1% DMSO; Sigma-Aldrich); GSK2193874 (GSK219, 10 μg, 25 μg in 2% DMSO and 5% Tween 80; Sigma-Aldrich); recombinant mouse Lipocalin-2 protein (LCN2, 1 μg in 10 μL PBS R&D Systems).

### In vitro Ca^2+^ imaging.

Primary cultured microglia were replated at 1 × 10^5^ cells per well for at least 2 hours on multi-well glass-bottom dishes (Thermo Fisher Scientific) coated with 10 μg/mL poly l-lysine. Cells were loaded with Fura 2-AM (4 μM; Life Technologies) for 1 hour and subsequently washed and imaged in standard bath solution of 140 mM NaCl, 5 mM KCl, 2 mM CaCl_2_, 2 mM MgCl_2_, and 10 mM HEPES, pH 7.4. An inverted Nikon Ti-E microscope was used for ratiometric Ca^2+^ imaging and fluorescence images were obtained with exposures of 0.2 ms at 340 nm and 0.2 ms at 380 nm excitation wavelengths through NIS-Elements imaging software (Nikon). The changes in Ca^2+^ signal was measured using the ratio of the fluorescence intensity obtained at 340 and 380 nm.

### Ex vivo Ca^2+^ imaging.

The *Cx3cr1^CreER/+^*:tdTomato:GCaMP6s mice were used for Ca^2+^ imaging of microglia in the spinal cord. The spinal slice preparation was performed identically to the electrophysiological recordings. The slices were placed in a recording chamber and microglial Ca^2+^ signaling was measured using a Nikon 2-photon system equipped with a MaiTaiHP Ti:sapphire laser tuned to 900 nm for 2-photon excitation of GCaMP6s with the laser power set to the lowest level, approximately 25 mW. The chamber was perfused with a recording solution containing 120 mM NaCl, 2.5 mM KCl, 2 mM CaCl_2_, 1 mM MgCl_2_, 1.25 mM NaH_2_PO_4_, 26 mM NaHCO_3_, 25 mM D-glucose, and saturated by a mixture of 95% O_2_ and 5% CO_2_. Images were collected 30–100 μm below the slice surface at a resolution of 512 × 512 pixels using a ×16 objective (NA = 1.05) immersed in the recording solution. All drugs were applied by bath application. Image acquisition was performed using NIS-Elements imaging software and then imported into ImageJ (NIH). The tdTomato expression was used for selecting the region of interest (ROI), and ROI analysis was performed using the multi-measure plugin in Fiji (ImageJ). The change in fluorescence was expressed as a relative percentage change, Δ*F*/*F*% = 100 × (*F*_t_ – *F*_0_)/*F*_0_, where *F*_t_ is the fluorescence at time t and *F*_0_ is the baseline at the start of the experiment.

### In vivo 2-photon Ca^2+^ imaging.

The GCaMP6s-expressing *Vglut2^Cre/+^* mice were anesthetized with 2% isoflurane. The skin was incised to expose the T12–L3 vertebrae and to remove the paravertebral muscle. A mouse spinal adaptor (World Precision Instruments) was attached to the vertebrae and a laminectomy was performed from T13–L1. A custom-made well was sealed with silicone adhesive around the T12–L3 vertebrae to facilitate the maintenance of the exposed spinal cord in the artificial cerebrospinal fluid. 2-photon Ca^2+^ imaging was performed using a Nikon microscope with a ×16 water-immersion lens and a MaiTaiHP Ti:sapphire laser tuned at 900 nm for 2-photon excitation of GCaMP6s. During 2-photon imaging, mice were placed on a heating pad at 37°C with the laser power set to the lowest level, approximately 20 mW, to avoid phototoxicity. Ca^2+^ imaging was performed by scanning image stacks (512 × 512 pixels, 4 μm Z steps) collected at a depth of approximately 80 μm from the spinal surface using NIS-Elements imaging software before and after GSK101 (1 μM) with or without GSK219 (25 μg) i.t. injections. Images were imported into Fiji (ImageJ), and MultiStackReg plugin was used to correct motion artifacts. The tdTomato expression was used for selecting ROI, and ROI analysis was performed using the multi-measure plugin in Fiji. The change in fluorescence was expressed as a relative percentage change, Δ*F/F*% = 100 × (*F*_t_ – *F*_0_)/*F*_0_, where *F*_t_ is the fluorescence at time t and *F*_0_ is the baseline at the start of the experiment.

### Spinal slice preparation and patch-clamp electrophysiology.

For spinal cord slice preparations, 6- to 8-week-old mice were deeply anesthetized with 2% isoflurane and transcardially perfused with preoxygenated ice-cold sucrose-based artificial cerebrospinal fluid containing 80 mM NaCl, 2.5 mM KCl, 1.25 mM NaH_2_PO_4_, 0.5 mM CaCl_2_, 3.5 mM MgCl_2_, 25 mM NaHCO_3_, 75 mM D-sucrose, 1.3 mM sodium ascorbate, and 3.0 mM sodium pyruvate. The L4–L5 spinal segment was placed in an agarose block and then cut on a vibratome (Leica). Spinal slices of 300 μm were then incubated in a recording solution containing 120 mM NaCl, 2.5 mM KCl, 2 mM CaCl_2_, 1 mM MgCl_2_, 1.25 mM NaH_2_PO_4_, 26 mM NaHCO_3_, and 25 mM D-glucose for approximately 30 minutes at 32°C. The recording solution was saturated by a mixture of 95% O_2_ and 5% CO_2_. Slices were maintained at room temperature for at least 30 minutes before being transferred into a recording chamber and perfused with oxygenated recording solution at 5 mL/minute during electrophysiologic recordings.

Whole-cell patch-clamp recording was used to record electrical and synaptic activities of the spinal lamina II neurons at 37°C. The resistance of the pipette electrode was 5–8 MΩ when filled with an internal solution containing 120 mM K-gluconate, 20 mM KCl, 2 mM MgCl_2_, 2 mM Na_2_-ATP, 0.5 mM Na_2_-GTP, 20 mM HEPES, and 0.5 mM EGTA (pH 7.3; 290–310 mOsm). The membrane current and AP firing were processed using a MultiClamp 700B amplifier (Axon Instruments). Signals were filtered at 2 kHz — lowpass filter frequency — and digitized at 10 kHz — sampling rate — with a Digidata 1440 A digitizer (Axon Instruments). In general, a seal resistance of greater than 2 GΩ within the patch stage and an access resistance of less than 35 MΩ within the cell stage were acceptable. Under voltage-clamp mode at a holding potential of −70 mV, sEPSCs were recorded in the presence of 100 μM picrotoxin (Tocris) and 1 μM strychnine (Sigma-Aldrich) to block GABA and glycine receptors, respectively. In current-clamp mode, a ramp protocol of depolarizing current (from 0–20 pA for 500 ms) was applied to assess the rheobase, RMP, and threshold. A step protocol of depolarizing currents (10 pA steps from 0 pA to 100 pA for 200 ms, or 40 pA steps from 40 pA to 120 pA for 500 ms) was applied to assess the number of AP firings. Data were collected using pClamp10 software (Axon Instruments) and then analyzed and plotted with Clampfit 10.7 (Axon Instruments) and Igor Pro 6.02 software (WaveMetrics).

The spinal microglia labeled with GFP or tdTomato were recorded under whole-cell patch-clamp mode. The resistance of the pipette electrode was 8–10 MΩ when filled with an internal solution containing 140 mM CsCl, 1 mM MgCl_2_, 0.5 mM EGTA, and 10 mM HEPES (pH 7.3; 310–320 mOsm). The membrane currents were evoked by voltage ramping from –100 to +100 mV for 500 ms at a holding potential of –10 mV. Data were analyzed and plotted with Clampfit 10.7 software.

### Statistics.

All values are reported as the mean ± SEM. Sample sizes were chosen based on the sample size determination (60), analysis of relevant prior studies and our pilot studies, and considerations including technical restraints, resource availability, and ethics of animal use. Unless otherwise defined, *n* numbers in figure legends represent biological replicates, or the number of mice, and no data point or result from a successful experiment was excluded from the analysis. Unpaired or paired 2-tailed *t* tests were used for comparison between 2 groups. 1-way ANOVA followed by Bonferroni’s post hoc tests were used for comparison among multiple groups. Differences between groups were considered statistically significant if *P* < 0.05. Statistical details and reported *n* values were provided in figure legends. All statistical testing was performed using Prism 8 (GraphPad).

### Study approval.

All of the animal experimental procedures were done according to the guidelines of the International Association for the Study of Pain and the NIH, and were approved by the IACUC of Washington University School of Medicine.

## Author contributions

HH and XH designed the experiments and wrote the manuscript. XH, LD, and SL performed the experiments and analyzed the data. ZL, PLW, and AMVH assisted with flow cytometry. KZ and XY assisted with virus injection and behavior assays. YZ and ZX assisted with calcium imaging and PCR assays. JF and LC assisted with genetic mouse line generation. KJL donated mice. JF, VKS, YW, RWG, and GFW assisted with manuscript preparation. HH and GFW supervised the project.

## Supplementary Material

Supplemental data

## Version 1 01/26/2023

In-Press Preview

## Version 2 03/01/2023

Electronic publication
