# Supplementary material for: A TRPV4-dependent neuroimmune axis in the spinal cord promotes neuropathic pain
Source: J Clin Invest. 2023 Mar 1;133(5):e161507. doi: 10.1172/JCI161507 (PMC9974096; doi:10.1172/JCI161507)
Supplement: Supplemental data [file jci-133-161507-s098.pdf]

Supplemental information for

A TRPV4-dependent neuro-immune axis in the spinal cord promotes neuropathic pain

Xueming Hu,[1#] Lixia Du,[1#] Shenbin Liu,[1#] Zhou Lan,[1] Kaikai Zang,[1] Jing Feng,[1] Yonghui Zhao,[1] Xingliang Yang,[1] Zili Xie,[1] Peter L. Wang,[2] Aaron M. Ver Heul,[1] Lvyi Chen,[1] Vijay K. Samineni,[1] Yanqing Wang,[3] Kory J. Lavine,[4] Robert W. Gereau IV,[1] Gregory F. Wu,[2,5]* and Hongzhen Hu[1]*

[1]Department of Anesthesiology, Center for the Study of Itch and Sensory Disorders, and Washington University Pain Center, Washington University School of Medicine, St. Louis, MO 63110, USA

[2]Department of Pathology and Immunology, Washington University School of Medicine, St. Louis, MO 63110, USA

[3]Institute of Acupuncture and Moxibustion, Institutes of Integrative Medicine; Department of Integrative Medicine and Neurobiology, School of Basic Medical Science; State Key Laboratory of Medical Neurobiology, Institutes of Brain Science, Fudan University, Shanghai 200032, China

[4]Department of Internal Medicine, Cardiovascular Division, Washington University School of Medicine, St. Louis, MO 63110, USA

[5]Department of Neurology, Washington University School of Medicine, St. Louis, MO 63110, USA

[#]**Authorship note:** XH, LD, and SL contributed equally to this work.

***Correspondence:**

Hongzhen Hu (hongzhen.hu@wustl.edu), Department of Anesthesiology, Center for the Study of Itch and Sensory Disorders, Washington University School of Medicine in St. Louis, 660 S. Euclid Avenue, St. Louis, MO 63110, Tel: 314-747-4317.

Gregory F. Wu (gfwu@wustl.edu), Department of Neurology, Washington University School of Medicine in St. Louis, 4523 Clayton Avenue, St. Louis, MO 63110, Tel: 314-362-3293.

Conflict of interest: The authors have declared that no conflict of interest exists.

This PDF file includes:

Supplemental Methods

Supplemental Figures 1 to 13

Supplemental Tables 1 to 2

Supplemental methods

Chemical ablation of $TRPV1^+$ sensory nerves

A capsaicin analog Resiniferatoxin (RTX, R8756, sigma) was subcutaneously injected into the neck of 8-week-old mice in three escalating (30, 70, and 100 µg/kg) doses on three consecutive days. Mice were then allowed to rest for 4 weeks before behavioral tests.

von Frey testing

Mechanical allodynia was represented as paw mechanical withdrawal threshold in response to von Frey filament (Bio-VF-M, Bioseb) stimulation. In brief, after 3 consecutive days of habituation, mice were placed in a plexiglass chamber on a wire net floor and allowed 10 to 15 min to habituate before a series of filaments (0.02, 0.04, 0.07, 0.16, 0.4, 0.6, 1.0, and 1.4 g) were applied to the hind paw with sustaining pressure to bend the filament for 5 s or elicit a paw withdrawal reflex within 5 s. Dixon's up-down method was used to determine the withdrawal threshold (1).

Accelerating rotarod test

Mouse motor coordination was assessed by rotarod testing (2). On the first two days, mice were placed on a rotarod apparatus (Ugo Basile) with an accelerating protocol (5-45 rpm for 5 min) and trained to maintain their belaying walking. On the third day, mice were tested three times with a 10-min interval. The average latency to fall off was recorded for analysis.

Open field test

Mouse locomotor activity was assessed by open field testing (2). Mice were placed at the center of a 25 cm (width) × 25 cm (length) × 25 cm (height) chamber and locomotor activities were monitored for 10 min by an overhead camera. The total distance moved and the duration of immobilization were analyzed by a tracking software (EthoVision, Noldus).

CatWalk analysis

A CatWalk XT system (Noldus) was used for Gait analysis based on the voluntary movement of mice in an enclosed walkway. The mouse was placed in the open end of the enclosed glass platform under a red ceiling light-emitting diode light and allowed to walk voluntarily through the walkway. While the mouse walked across the glass floor, a high-speed camera positioned underneath the apparatus captured images of the illuminated area of each paw and transferred the data to the gait analysis software (CatWalk XT, version 10.0; Noldus). A minimum of three serial step cycles, or complete passes through the tunnel, were gathered as valid data. In this study, three available parameters were identified to evaluate dynamic behaviors associated with pain-related behaviors (3): (1) "swing" as the duration of no hind paw contact with the glass plate; (2) "single stance" as the duration of ground contact of a single hind paw where the contralateral hind paw did not touch the glass plate; and (3) "duty cycle" as a percentage of Step Cycle: Duty Cycle = Stand/(Stand + Swing) × 100%. Data were calculated as the percentage of ipsilateral (right)/contralateral (left) hind paw.

Intraspinal injection of AAV vector

Mice were anesthetized under isoflurane anesthesia on an isothermal heating pad. The dorsal surface above the thoracic and lumbar spine was shaved and cleaned. T12-L3 vertebrae was exposed after a 3-cm midline incision was made on the dorsal surface. The paraspinal muscles were separated to expose the T13/L1 and L1/L2 intervertebral foramen. Two separate 400 nl of AAV-EF1a-DIO-GCaMP6s-P2A-nls-tdTomato viruses (51082-AAV1, addgene, 5×10^{12} vg/ml), AAV-hSyn-EGFP (105540-AAV9, addgene, 1×10^{13} vg/mL), or AAV-hSyn-EGFP-Cre (50465-AAV9, addgene, 1×10^{13} vg/mL) in 60 nl/min were injected into the surface of the spinal dorsal horn (mediolateral: 400 mm; dorsoventral: 300 mm) using glass micropipettes connected to the Nanoliter Injector (World Precision Instruments). After microinjection, the muscles and skin were sutured with 6-0 silk, and mice were recovered for 3 weeks to allow stable transgene expression.

Primary spinal microglia culture

Primary culture of mouse spinal cord microglia was prepared as described (4). Briefly, spinal cord tissues from newborn mice were dissected and the meninges were removed. The tissues were incubated in trypsin/EDTA solution (Invitrogen) at 37°C for 10 min and triturated with a 1 ml pipette and subsequently filtered through a 40 µm mesh cell strainer to remove tissue clumps after stopping the enzymatic digestion. The dispersed cells were subjected to centrifugation (5 min, 2000 rpm) and resuspended in DMEM containing 10% fetal bovine serum (FBS) and penicillin (100 U/ml)/streptomycin (100 µg/ml). The mixed glial cell solution was plated in 6-well plates and incubated in a humidified culture incubator (37°C, 5% CO_2). Non-adhering cells were removed 24 h after plating and the culture medium was replaced. The conditioned media in each well was replaced with fresh media every 3 days. The 6-well plates were shaken at 200 rpm for 4 hours after two to three weeks of plating and the media was collected, counted, and then cultured (1×10^5 cells per ml density) in a serum-free medium for 24 h before experiments. The ratio of microglia in the mixed glial cell cultures was determined by immunofluorescence, and approximately 100% of the cells were IBA1-positive, indicating that the majority of the cells were microglia.

Flow cytometry

Spinal dorsal horn tissues from the *Trpv4*^{eGFP} mice were collected and digested in trypsin/EDTA solution (Invitrogen) at 37°C for 10 min, followed by trituration using a 1 ml pipette and subsequently filtered through a 40 µm mesh cell strainer. The dispersed cells were centrifuged at 2000 rpm for 5 min and then stained with Zombie UV viability dye (1:500, Biolegend) according to the manufacturer's instructions for 20 min at room temperature. After being washed with FACS buffer (PBS containing 2% FBS), the cells were stained with a standard panel of immunophenotyping antibodies (1:300, listed in Supplemental Table 1), on ice for 30 min. Compensation was performed at the beginning of each experiment. Data of samples were collected using BD Fortessa X-20 flow cytometer (BD Biosciences) and analyzed using FlowJo software (V10, Tree Star). Microglia were defined as live cells with $CD45^+$ $CD11b^+$ $CCR2^-$ expression pattern.

Immunofluorescence and analysis

Mice were deeply anesthetized with 2% isoflurane and were perfused intracardially with 0.01 M phosphate-buffered saline (PBS) and 4% paraformaldehyde (PFA) in 0.1 M phosphate buffer (PB). L4-5 spinal tissues were removed, post fixed in 4% PFA overnight at 4°C, then transferred to 30% sucrose in 0.1 M PB for 3-5 days at 4°C. 35-µm-thick serial sections were prepared from the tissues using a cryostat and blocked with 10% normal donkey serum in 0.01 M PBS containing 0.3% Triton X-100 for 1 hour at room temperature. Spinal sections were then incubated for 48 h at 4°C with primary antibodies (1:1000; listed in Supplemental Table 1). After washing with PBS, free-floating sections were incubated for 1 h at room temperature with the corresponding Alexa Fluor 405, 488-, or 594-conjugated secondary antibodies (1:1000; listed in Supplemental Table 1). After mounting on slides, sections were sealed in mounting medium with or without DAPI (Fluoromount-G; SouthernBiotech) for storage and visualization. Fluorescence images were captured using a Nikon A1R confocal microscope, and images are shown as merged Z-stack projections consisting of approximately 15 optical slices (1 µm per slice). Measurements of the number of TRPV4-$eGFP^+$, $CD31^+$, $IBA1^+$ $CX3CR1^+$, $Tmem119^+$, and EdU^+ cells and the mean fluorescence intensity (MFI) of $CX3CR1^+$, $Tmem119^+$, and $IBA1^+$ signals in the L4-5 spinal dorsal horn were obtained using Fiji (ImageJ, http://fiji.sc) with the default parameters.

For the microglia proliferation experiment, 5-ethynyl-2′-deoxyuridine (EdU, 10 mM, 10 ml/kg, 900584, Sigma) was i.p. injected once per day for 3 consecutive days in mice. EdU staining was performed using Click-iT™ EdU cell proliferation kits for imaging (C10337/C10339, Invitrogen), based on manufacturer's instructions.

For the dendritic spine analysis, biocytin (0.25%, 3349, Tocris) was included in the internal solution, and neurons were filled for 15-20 min in the whole-cell patch-clamp configuration. The biocytin-labeled neurons were stained by streptavidin with Alexa Fluor™ 488 conjugate (S11223, Invitrogen), and then

captured using an Olympus FV1200 confocal microscope with 100× oil objective. Imaris software 9.0.1 (Bitplane) was used to reconstruct the dendritic spines and analyzed the spine density with the default parameters.

Real-time quantitative PCR

Mice were perfused intracardially with PBS under deep anesthesia. Total RNA from the ipsilateral side of L4-5 spinal dorsal horn tissues was extracted using TRIzol reagent (Invitrogen) according to manufacturer's instructions. 1 µg of total RNA was reverse transcribed into cDNA using Maxima™ H Minus cDNA Synthesis Master Mix (M1682; Thermo Scientific). Each process was carried out in triplicate with a 20-µl reaction mixture (containing 2 µl of cDNA and 10 µM of gene-specific primers) using a Fast SYBR™ Green Master Mix (4385612; Thermo Scientific). Quantitative PCR was run on a 7300 Plus Real-Time PCR system (Applied Biosystems) with thermocycling conditions of 95°C for 30 s followed by 40 amplification cycles (5 s at 95°C and 30 s at 60°C). The mRNA expression levels were analyzed using the $2^{-\Delta\Delta Ct}$ method and were normalized to the average of glyceraldehyde-3-phosphate dehydrogenase (*Gapdh*). The forward and reverse primers (Sigma) are listed in Supplemental Table 2.

RNAscope

RNA in situ hybridization (ISH) was performed using the RNAscope Multiplex Fluorescent Reagent Kit v2 (322300-USM; Advanced Cell Diagnostics) according to manufacturer's instructions. Briefly, 30-µm-thick spinal slices from $Cx3cr1^{CreER/+}$::tdTomato mice were prepared using a cryostat and incubated with hydrogen peroxide for 10 min at room temperature and then boiled with target retrieval reagent for 5 min. The slices were incubated with protease III at 40°C for 30 min and subsequently subjected to hybridization of the RNA probes to the mRNA targets in the HybEZ Oven for 2 hours at 40°C. The RNA probes (*Trpv4*, NM_022017.3, 406071, Advanced Cell Diagnostics) contained 20 pairs of probes (conceptualized as ZZ) complementary to the target mRNA. After hybridization, the spinal slices were processed for standard signal amplification and fluorophores staining steps.

Enzyme-linked immunosorbent assay (ELISA)

The concentrations of LCN2 in supernatant samples from cultured primary microglia were detected by ELISA according to manufacturer's instructions (MLCN20, R&D Systems). The plates were read at 450 nm and 570 nm using a microplate reader (Synergy H1 Hybrid Reader, BioTek), and the standard curve was included in each experiment.

References

1. Chaplan SR, Bach FW, Pogrel JW, Chung JM, and Yaksh TL. Quantitative assessment of tactile allodynia in the rat paw. Journal of neuroscience methods. 1994;53(1):55-63.
2. Donnelly CR, Jiang C, Andriessen AS, Wang K, Wang Z, Ding H, Zhao J, Luo X, Lee MS, Lei YL, et al. STING controls nociception via type I interferon signalling in sensory neurons. Nature. 2021;591(7849):275-80.
3. Hu XM, Yang W, Du LX, Cui WQ, Mi WL, Mao-Ying QL, Chu YX, and Wang YQ. Vascular Endothelial Growth Factor A Signaling Promotes Spinal Central Sensitization and Pain-related Behaviors in Female Rats with Bone Cancer. Anesthesiology. 2019;131(5):1125-47.
4. Tamashiro TT, Dalgard CL, and Byrnes KR. Primary microglia isolation from mixed glial cell cultures of neonatal rat brain tissue. Journal of visualized experiments: JoVE. 201266): e3814.

Supplemental figures

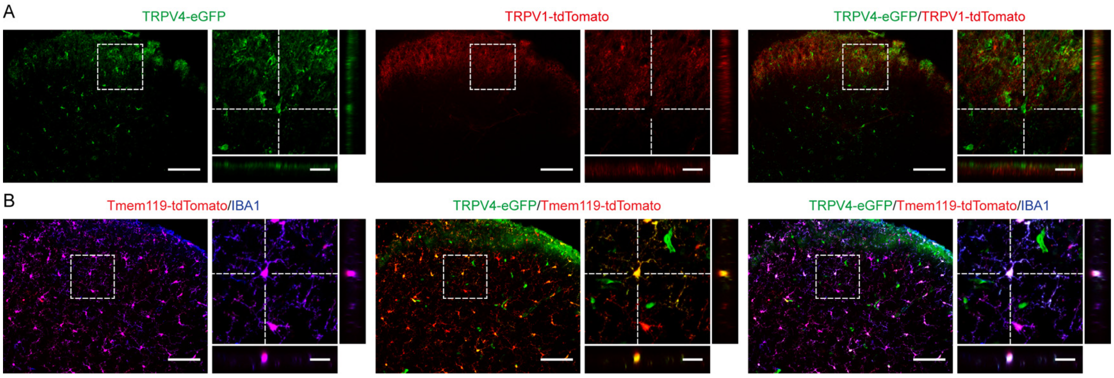

Supplemental Figure 1. TRPV4-eGFP is colocalized with Tmem119-tdTomato but not TRPV1-tdTomato in spinal cord dorsal horn. (**A**) Representative images of TRPV4-eGFP and TRPV1-tdTomato co-expression in the spinal cord dorsal horn of *Trpv4*^{eGFP}::*Trpv1*$^{Cre/+}$::tdTomato mice. (**B**) Representative images of TRPV4-eGFP, IBA1, and Tmem119-tdTomato co-expression in the spinal cord dorsal horn of *Trpv4*^{eGFP}::*Tmem119*$^{CreER/+}$::tdTomato mice. Tamoxifen was i.p. administrated for 5 consecutive days. Scale bar = 100 μm and 20 μm (zoom).

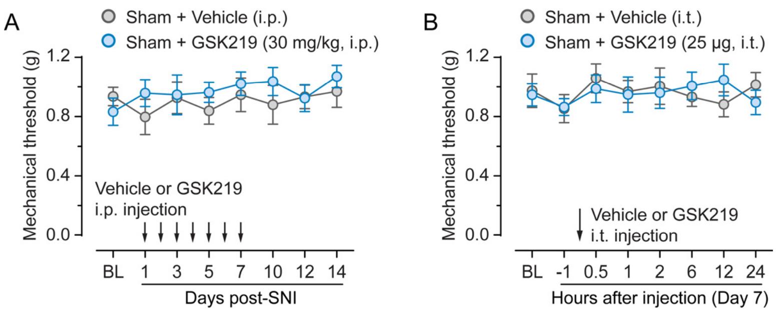

Supplemental Figure 2. Pharmacological inhibition of TRPV4 function by GSK219 does not affect baseline mechanical sensitivity. (**A**) Time course of paw withdrawal threshold (von Frey) following sham surgery in *wt* mice treated with repeated i.p. injection of vehicle or GSK219 (once per day from day 1 to 7 post-sham treatment). n = 8 mice per group. n.s. by two-way repeated ANOVA with *post hoc* Bonferroni test. (**B**) Time course of paw withdrawal threshold (von Frey) following sham surgery in *wt* mice treated with single i.t. injection of vehicle or GSK219 on day 7 post-sham treatment. n = 8 mice per group. n.s. by two-way repeated ANOVA with *post hoc* Bonferroni test.

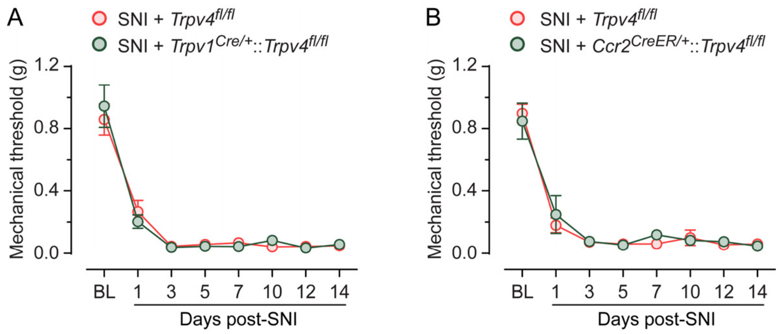

Supplemental Figure 3. Genetic ablation of TRPV4 function in TRPV1-expressing nociceptors or blood-derived CCR2-expressing monocytes do not affect mechanical nociception. (**A**) Time course of paw withdrawal threshold (von Frey) following SNI surgery in *Trpv4*$^{fl/fl}$ control littermates and *Trpv1*$^{Cre/+}$::*Trpv4*$^{fl/fl}$ mice. n = 6 mice per group. n.s. by two-way repeated ANOVA with *post hoc* Bonferroni test. (**B**) Time course of paw withdrawal threshold (von Frey) following SNI surgery in *Trpv4*$^{fl/fl}$ control littermates and *Ccr2*$^{CreER/+}$::*Trpv4*$^{fl/fl}$ mice. n = 8 mice per group. n.s. by two-way repeated ANOVA with *post hoc* Bonferroni test. Data are represented as mean ± s.e.m.

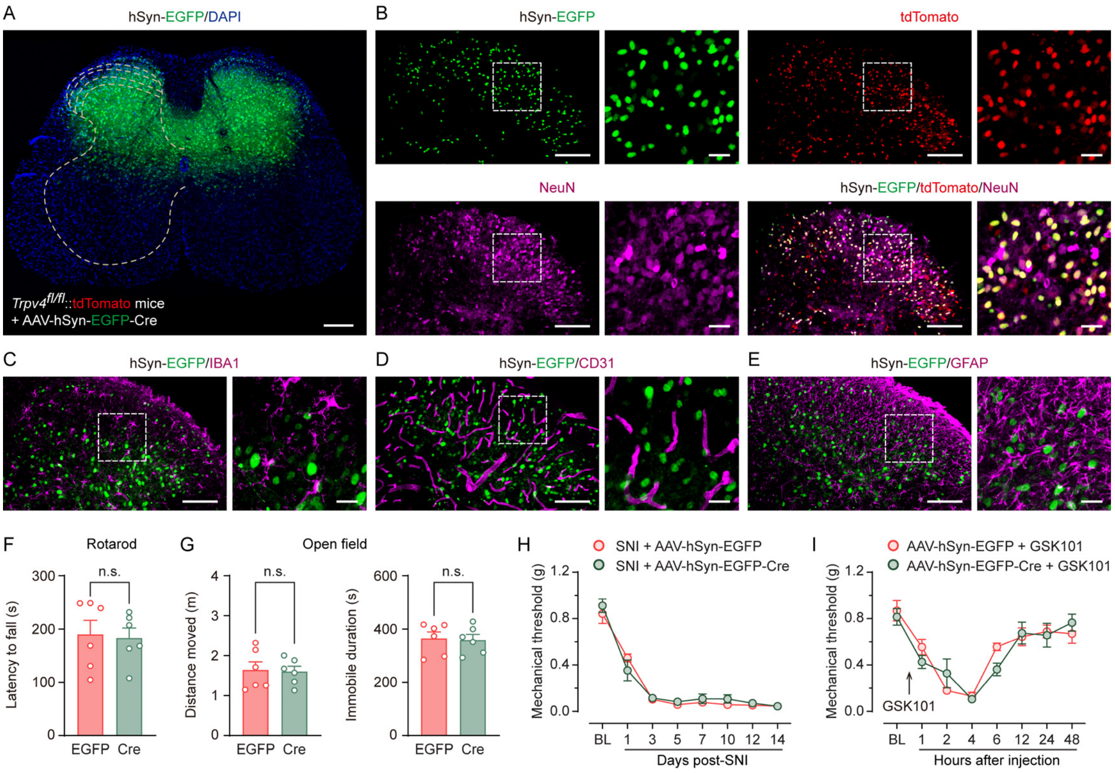

Supplemental Figure 4. The effects of neuron-specific *Trpv4* cKO on the mechanical allodynia and sensorimotor behaviors following SNI or GSK101 administration. (**A**) Representative image of infection of AAV-hSyn-EGFP-Cre in the spinal dorsal horn of *Trpv4*$^{fl/fl}$::tdTomato mice. Scale bars, 200 μm. (**B**) Representative images of hSyn-EGFP-expressing cells with tdTomato, and neuronal marker NeuN. (**C-E**) Representative images of hSyn-EGFP-expressing cells with microglial marker IBA1 (**C**), endothelial marker CD31 (**D**), and astrocytic marker GFAP (**E**) in the spinal dorsal horn. Scale bars = 100 μm and 20 μm (zoom). (**F**) No differences were observed in motor coordination (Rotarod) between control AAV-hSyn-EGFP-treated and AAV-hSyn-EGFP-Cre-treated mice in the accelerating rotarod test to measure motor function (5 min test duration). n = 6 mice per group. n.s. by unpaired Student's t-test. (**G**) No differences were observed in locomotor activity (Open field) between control AAV-hSyn-EGFP-treated and AAV-hSyn-EGFP-Cre-treated mice in the open field test to measure locomotor activity (10 min test duration). n = 6 mice per group. n.s. by unpaired Student's t-test. (**H**) Time course of paw withdrawal threshold (von Frey) following SNI surgery in control AAV-hSyn-EGFP-treated and AAV-hSyn-EGFP-Cre-treated mice. n = 6 mice per group. n.s. by two-way repeated ANOVA with *post hoc* Bonferroni test. (**I**) Time course of paw withdrawal threshold (von Frey) following i.t. injection of GSK101 in control AAV-hSyn-EGFP-treated and AAV-hSyn-EGFP-Cre-treated mice. GSK101 (1 μg) was i.t. injected 1 h after detecting the BL of paw withdrawal threshold. n = 6 mice per group. n.s. by two-way repeated ANOVA with *post hoc* Bonferroni test. Data are represented as mean ± s.e.m.

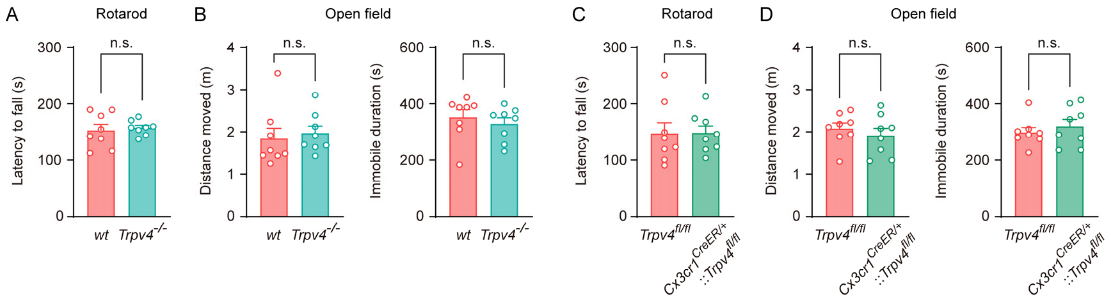

Supplemental Figure 5. Genetic ablation of TRPV4 function does not affect sensorimotor functions. (**A**) No differences were observed in motor coordination (Rotarod) between *wt* mice and *Trpv4*$^{-/-}$ mice in the accelerating rotarod test to measure motor function (5 min test duration). n = 8 mice per group. n.s. by unpaired Student's t-test. (**B**) No differences were observed in locomotor activity (Open field) between *wt* mice and *Trpv4*$^{-/-}$ mice in the open field test to measure locomotor activity (10 min test duration). n = 8 mice per group. n.s. by unpaired Student's t-test. (**C**) No differences were observed between *Trpv4*$^{fl/fl}$ control littermates and *Cx3cr1*$^{CreER/+}$::*Trpv4*$^{fl/fl}$ mice in the accelerating rotarod test to measure motor function (5 min test duration). n = 8 mice per group. n.s. by unpaired Student's t-test. (**D**) No differences were observed between *Trpv4*$^{fl/fl}$ control littermates and *Cx3cr1*$^{CreER/+}$::*Trpv4*$^{fl/fl}$ mice in the open field test to measure locomotor activity (10 min test duration). n = 8 mice per group. n.s. by unpaired Student's t-test. Data are represented as mean ± s.e.m.

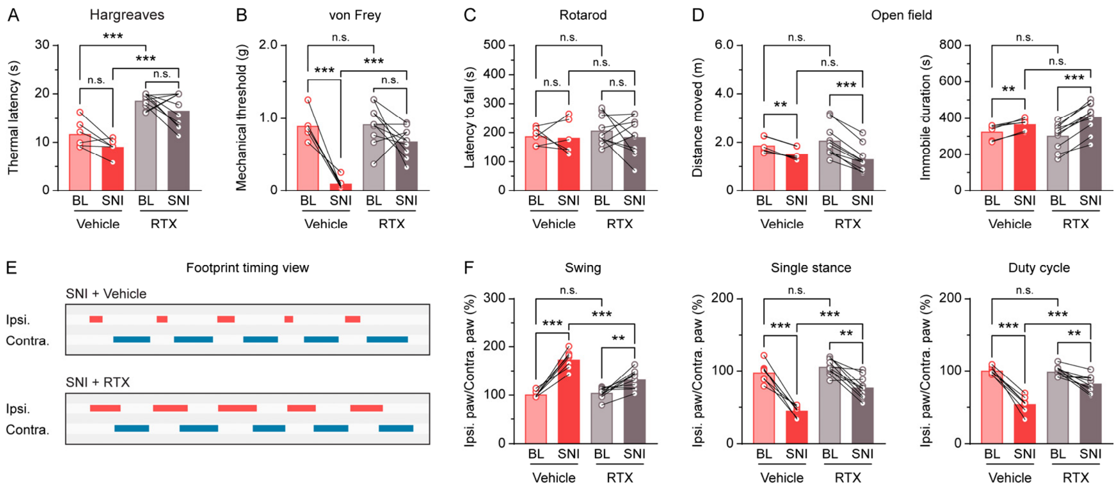

Supplemental Figure 6. The effects of chemically induced ablation of TRPV1-expressing nociceptors using RTX on the mechanical allodynia, sensorimotor behaviors, and abnormal gaits following SNI. (**A**) Thermal withdrawal latency (Hargreaves) following SNI surgery in *wt* mice treated with s.c. injection of vehicle or RTX on day 7 post-SNI. n = 6-10 mice per group. ***p < 0.001 by unpaired Student's t-test. (**B**) Paw withdrawal threshold (von Frey) following SNI surgery in *wt* mice treated with s.c. injection of vehicle or RTX on day 7 post-SNI. n = 6-10 mice per group. ***p < 0.001 by paired or unpaired Student's t-test. (**C**) Motor coordination (Rotarod) was detected following SNI surgery in *wt* mice treated with s.c. injection of vehicle or RTX on day 7 post-SNI in the accelerating rotarod test to measure motor function (5 min test duration). n = 6-10 mice per group. n.s. by paired or unpaired Student's t-test. (**D**) Locomotor activity (Open field) was detected following SNI surgery in *wt* mice treated with s.c. injection of vehicle or RTX on day 7 post-SNI in the open field test to measure locomotor activity (10 min test duration). n = 6-10 mice per group. **p < 0.01, ***p < 0.001 by paired Student's t-test. (**E** and **F**) Representative footprint timing view and CatWalk gait analysis of *wt* mice treated with s.c. injection of vehicle or RTX on day 7 post-SNI. n = 7-10 mice per group. **p < 0.01, ***p < 0.001 by paired or unpaired Student's t-test. Data are represented as mean ± s.e.m.

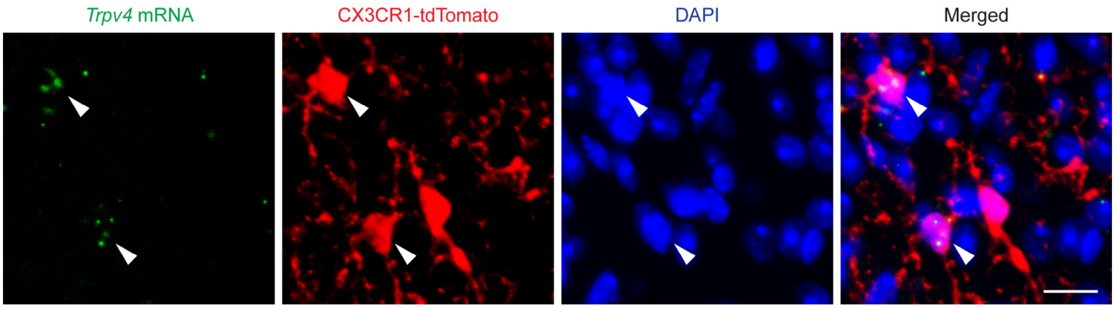

Supplemental Figure 7. The expression of *Trpv4* mRNA in spinal microglia. Representative RNAscope images of *Trpv4* mRNA with CX3CR1-tdTomato in the spinal dorsal horn of *Cx3cr1*$^{CreER/+}$::tdTomato mice on day 7 post-SNI. Tamoxifen was i.p. administrated for 5 consecutive days 4 weeks before SNI surgery. Scale bar = 10 μm.

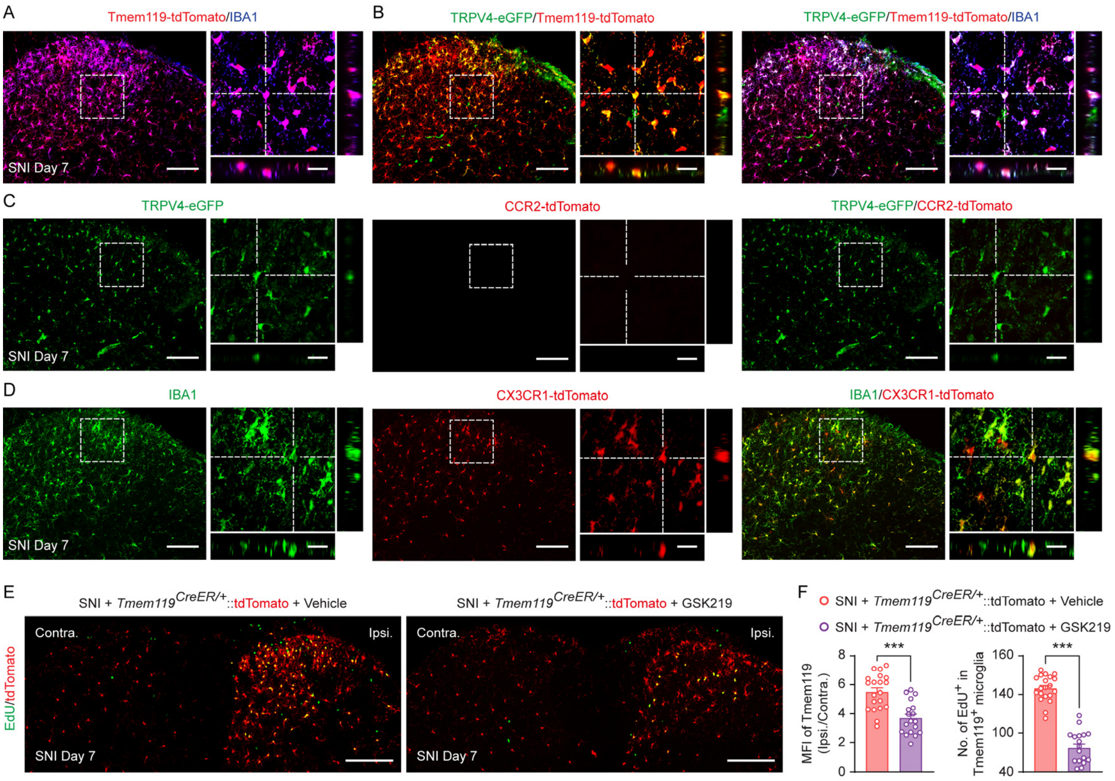

Supplemental Figure 8. TRPV4 is expressed by microglia-specific marker Tmem119 but not monocytic marker CCR2, and mediates the activation and proliferation of Tmem119^{+} microglia. (**A** and **B**) Representative images of TRPV4-eGFP, Tmem119-tdTomato, and IBA1 co-expression in the spinal dorsal horn of *Trpv4*^{eGFP}::*Tmem119*$^{CreER/+}$::tdTomato mice on day 7 post-SNI. (**C**) Representative images of TRPV4-eGFP and CCR2-tdTomato co-expression in the spinal cord dorsal horn of *Trpv4*^{eGFP}::*Ccr2*$^{CreER/+}$::tdTomato mice on day 7 post-SNI. (**D**) Representative images of IBA1 and CX3CR1-tdTomato co-expression in the spinal cord dorsal horn of *Cx3cr1*$^{CreER/+}$::tdTomato mice on day 7 post-SNI. Tamoxifen was i.p. administrated for 5 consecutive days 4 weeks before SNI surgery. Scale bar = 100 μm and 20 μm (zoom). (**E** and **F**) Representative images of Tmem119-tdTomato and EdU co-expression in spinal dorsal horn (**E**) and quantification of the mean fluorescence intensity (MFI) of Tmem119-tdTomato and number of EdU^{+}/Tmem119-tdTomato^{+} microglia from *Tmem119*$^{CreER/+}$::tdTomato mice treated with GSK219 on day 7 post-SNI (**F**). GSK219 was i.p. administrated once per day for 7 consecutive days starting on day 1 post-SNI; while EdU was i.p. administrated once per day for 3 consecutive days starting on day 1 post-SNI. n = 18-20 spinal slices from 4 mice per group. ***$p < 0.001$ by unpaired Student's t-test. Scale bar = 200 μm. Data are represented as mean ± s.e.m.

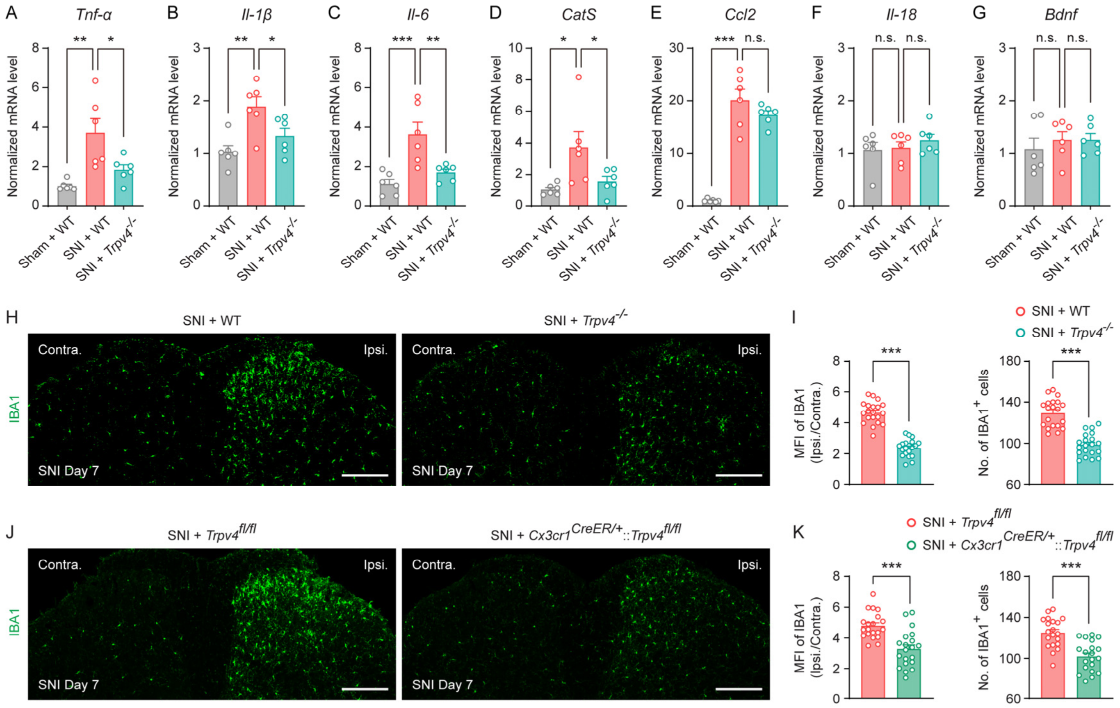

Supplemental Figure 9. Genetic ablation of TRPV4 function reduces SNI-induced production of microglia-derived proinflammatory cytokines and IBA1 expression in the spinal cord. (**A-G**) RT-qPCR quantification of the mRNA expression of *Tnf-α* (**A**), *Il-1β* (**B**), *Il-6* (**C**), *CatS* (**D**), *Ccl2* (**E**), *Il-18* (**F**), and *Bdnf* (**G**) in the spinal cord from *wt* mice and $Trpv4^{-/-}$mice on day 7 after sham or SNI surgery. n = 6 mice per group. *$p < 0.05$, **$p < 0.01$, ***$p < 0.001$ by one-way ANOVA with *post hoc* Bonferroni test. (**H** and **I**) Representative images of IBA1 expression in the spinal dorsal horn of *wt* mice and $Trpv4^{-/-}$ mice on day 7 post-SNI (**H**). Quantification of MFI of IBA and number of IBA1$^+$ microglia in the spinal cord of *wt* mice and $Trpv4^{-/-}$mice on day 7 post-SNI (**I**). n = 20 spinal slices from 4 mice per group. ***$p < 0.001$ by unpaired Student's t-test. Scale bar = 200 µm. (**J** and **K**) Representative images of IBA1 expression in the spinal dorsal horn of $Trpv4^{fl/fl}$ control littermates and $Cx3cr1^{CreER/+}$::$Trpv4^{fl/fl}$ mice on day 7 post-SNI (**J**). Quantification of MFI of IBA1 and number of IBA1$^+$ microglia in the spinal cord of $Trpv4^{fl/fl}$ control littermates and $Cx3cr1^{CreER/+}$::$Trpv4^{fl/fl}$ mice on day 7 post-SNI (**K**). n = 20 spinal slices from 4 mice per group. ***$p < 0.001$ by unpaired Student's t-test. Tamoxifen was i.p. administrated for 5 consecutive days 4 weeks before SNI surgery. Scale bar = 200 µm. Data are represented as mean ± s.e.m.

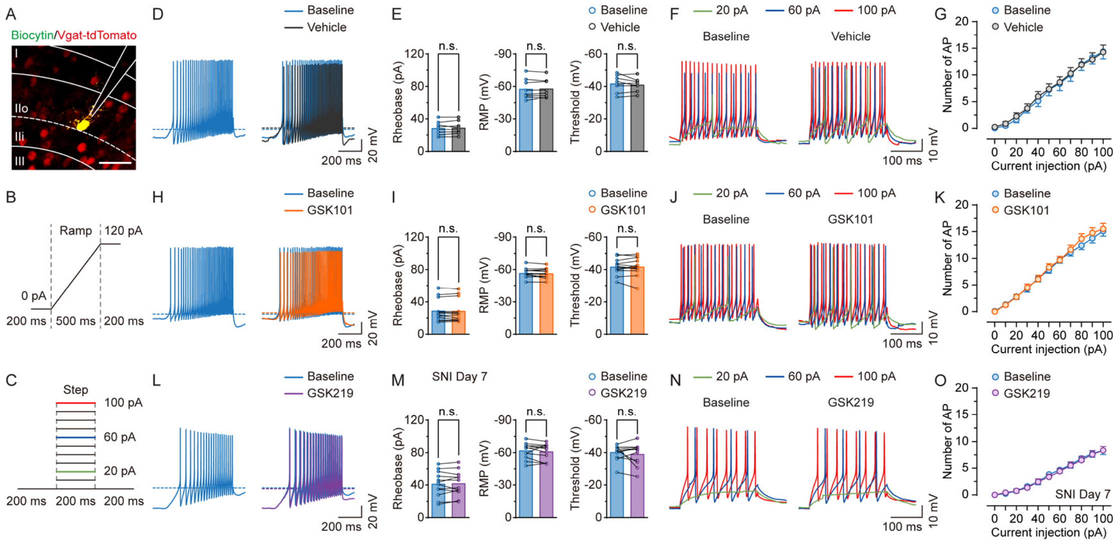

Supplemental Figure 10. Activation of TRPV4 does not affect the excitability of inhibitory interneurons in the spinal cord. (**A**-**C**) Representative recorded Vgat-tdTomato^{+} neuron in the lamina IIo of spinal cord slice (**A**), ramp protocol of depolarizing current applied to assess the rheobase, RMP and threshold (**B**), and step protocol of depolarizing currents applied to assess the number of AP firings (**C**). (**D**-**G**) Representative traces and quantification of the rheobase, RMP, and threshold of current ramp-induced AP firings (**D** and **E**), as well as representative traces and quantification of the number of current step-induced AP firings (**F** and **G**) in spinal lamina IIo neurons from *Vgat*$^{Cre/+}$::tdTomato mice with vehicle perfusion. n = 8 neurons from 3 mice. n.s. by paired Student's t-test (**E**), n.s. by two-way repeated ANOVA with *post hoc* Bonferroni test (**G**). (**H**-**K**) Representative traces and quantification of the rheobase, RMP, and threshold of current ramp-induced AP firings (**H** and **I**), as well as representative traces and quantification of the number of current step-induced AP firings (**J** and **K**) in spinal lamina IIo neurons from *Vgat*$^{Cre/+}$::tdTomato mice with GSK101 (300 nM) perfusion. n = 12 neurons from 3 mice. n.s. by paired Student's t-test (**I**), n.s. by two-way repeated ANOVA with *post hoc* Bonferroni test (**K**). (**L**-**O**) Representative traces and quantification of the rheobase, RMP, and threshold of current ramp-induced AP firings (**L** and **M**), as well as representative traces and quantification of the number of current step-induced AP firings (**N** and **O**) in spinal lamina IIo neurons from *Vgat*$^{Cre/+}$::tdTomato mice with GSK219 (300 nM) perfusion on day 7 post-SNI. n = 12 neurons from 3 mice. n = 12 neurons from 3 mice. n.s. by paired Student's t-test (**M**), n.s. by two-way repeated ANOVA with *post hoc* Bonferroni test (**O**). Data are represented as mean ± s.e.m.

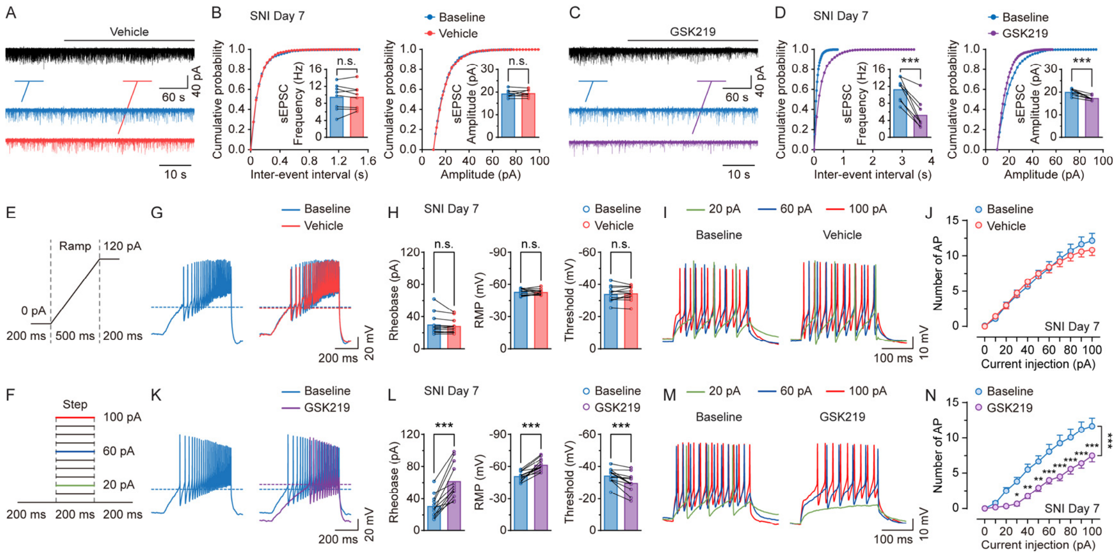

Supplemental Figure 11. Acute application of TRPV4 antagonist GSK219 suppresses SNI-induced neuronal hyperactivity in spinal lamina IIo neurons. (**A** and **B**) Representative traces and quantification of the frequency and amplitude of sEPSCs in spinal lamina IIo neurons from *Vglut2*$^{Cre/+}$::tdTomato mice with vehicle perfusion on day 7 post-SNI. n = 8 neurons from 3 mice per group. n.s. by paired Student's t-test. (**C** and **D**) Representative traces and quantification of the frequency and amplitude of sEPSCs in spinal lamina IIo neurons from *Vglut2*$^{Cre/+}$::tdTomato mice with GSK219 (300 nM) perfusion on day 7 post-SNI. n = 8 neurons from 3 mice per group. ***$p < 0.001$ by paired Student's t-test. (**E** and **F**) Ramp protocol of depolarizing current was applied to assess the rheobase, RMP, and threshold (**E**), and step protocol of depolarizing currents was applied to assess the number of AP firings (**F**). (**G**-**J**) Representative traces and quantification of the rheobase, RMP, and threshold of current ramp-induced AP firings (**G** and **H**), as well as representative traces and quantification of the number of current step-induced AP firings (**I** and **J**) in spinal lamina IIo neurons from *Vglut2*$^{Cre/+}$::tdTomato mice with vehicle perfusion. n = 12 neurons from 3 mice. n.s. by paired Student's t-test (**H**), n.s. by two-way repeated ANOVA with *post hoc* Bonferroni test (**J**). (**K**-**N**) Representative traces and quantification of the rheobase, RMP, and threshold of current ramp-induced AP firings (**K** and **L**), as well as representative traces and quantification of the number of current step-induced AP firings (**M** and **N**) in spinal lamina IIo neurons from *Vglut2*$^{Cre/+}$::tdTomato mice with GSK219 (300 nM) perfusion. n = 12 neurons from 3 mice. ***$p < 0.001$ by paired Student's t-test (L), *$p < 0.05$, **$p < 0.01$, ***$p < 0.001$ by two-way repeated ANOVA with *post hoc* Bonferroni test (**N**). Data are represented as mean ± s.e.m.

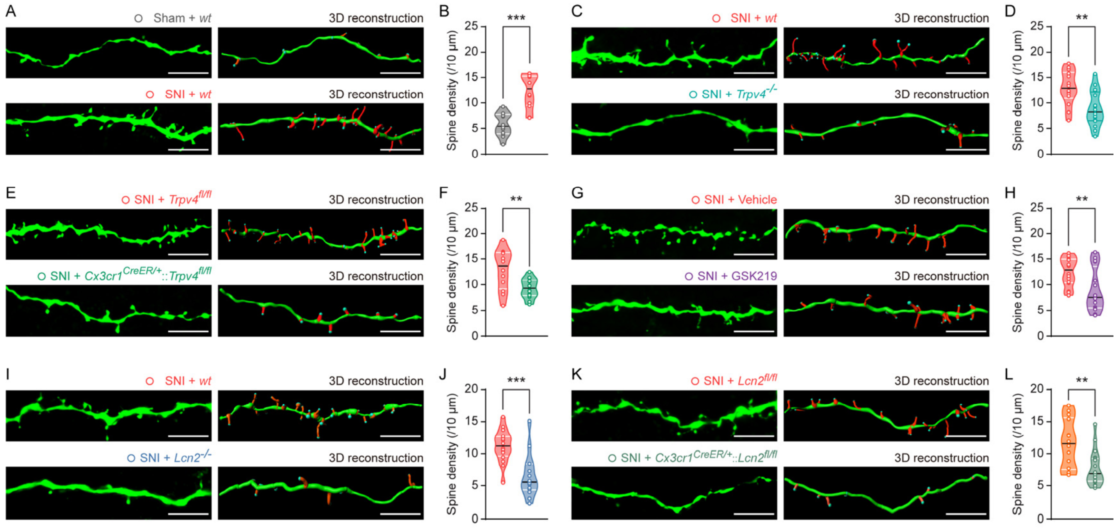

Supplemental Figure 12. Genetic or pharmacological inhibition of TRPV4 or LCN2 inhibits SNI-induced dendritic spine remodeling in spinal lamina IIo neurons. (**A** and **B**) Representative images with 3D reconstruction and quantification of the dendritic spine density in spinal lamina IIo neurons from *wt* mice on day 7 post-sham or SNI. n = 15-20 neurons from 3 mice per group. ***p < 0.001 by unpaired Student's t-test. Scale bar = 10 μm. (**C** and **D**) Representative images with 3D reconstruction and quantification of the dendritic spine density in spinal lamina IIo neurons from *wt* mice and $Trpv4^{-/-}$ mice on day 7 post-SNI. n = 14-16 neurons from 4 mice per group. **p < 0.01 by unpaired Student's t-test. Scale bar = 10 μm. (**E** and **F**) Representative images with 3D reconstruction and quantification of the dendritic spine density in spinal lamina IIo neurons from $Trpv4^{fl/fl}$ control littermates and $Cx3cr1^{CreER/+}$::$Trpv4^{fl/fl}$ mice on day 7 post-SNI. n = 12-14 neurons from 4 mice per group. **p < 0.01 by unpaired Student's t-test. Scale bar = 10 μm. (**G** and **H**) Representative images with 3D reconstruction and quantification of the dendritic spine density in spinal lamina IIo neurons from *wt* mice treated with vehicle or GSK219 (30 mg/kg, i.p., once per day from day 1 to 7 post-SNI) on day 7 post-SNI. n = 15-20 neurons from 3 mice per group. **p < 0.01 by unpaired Student's t-test. Scale bar = 10 μm. (**I** and **J**) Representative images with 3D reconstruction and quantification of the dendritic spine density in spinal lamina IIo neurons from *wt* mice and $Lcn2^{-/-}$ mice on day 7 post-SNI. n = 14-16 neurons from 4 mice per group. ***p < 0.001 by unpaired Student's t-test. Scale bar = 10 μm. (**K** and **L**) Representative images with 3D reconstruction and quantification of the dendritic spine density in spinal lamina IIo neurons from $Lcn2^{fl/fl}$ control littermates and $Cx3cr1^{CreER/+}$::$Lcn2^{fl/fl}$ mice on day 7 post-SNI. n = 14-18 neurons from 4 mice per group. **p < 0.01 by unpaired Student's t-test. Scale bar = 10 μm. Data are represented as mean ± s.e.m.

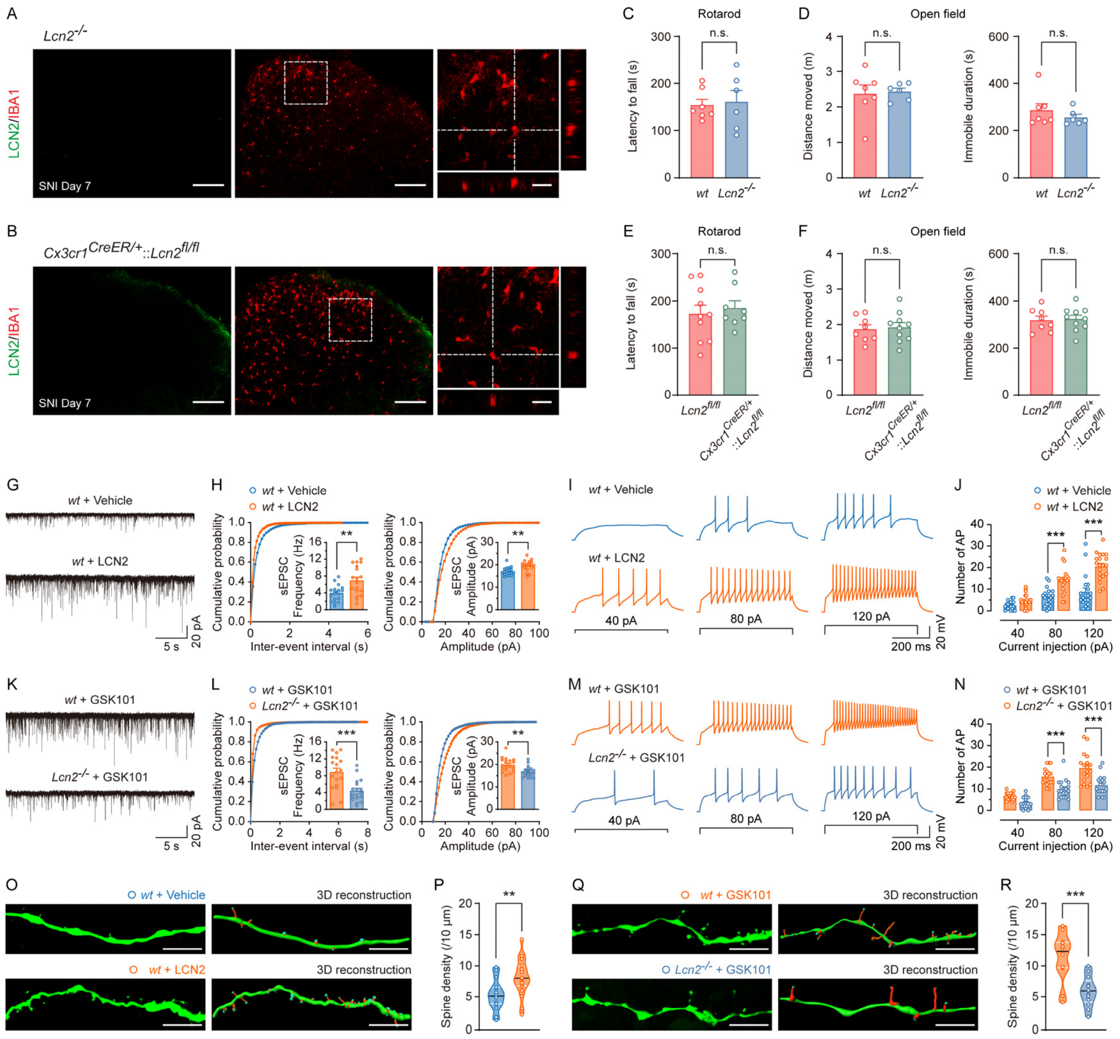

Supplemental Figure 13. LCN2 is necessary and sufficient to produce functional and structural plasticity of spinal excitatory neurons. (**A**) Representative images of LCN2 and IBA1 expression in the spinal dorsal horn of *Lcn2*$^{-/-}$ mice on day 7 post-SNI. Scale bar = 100 μm and 20 μm (zoom). (**B**) Representative images of LCN2 and IBA1 expression in the spinal dorsal horn of *Cx3cr1*$^{CreER/+}$::*Lcn2*$^{fl/fl}$ mice on day 7 post-SNI. Scale bar = 100 μm and 20 μm (zoom). (**C**) No differences were observed in motor coordination (Rotarod) between *wt* mice and *Lcn2*$^{-/-}$ mice in the accelerating rotarod test to measure motor function (5 min test duration). n = 8 mice per group. n.s. by unpaired Student's t-test. (**D**) No differences were observed in locomotor activity (Open field) between *wt* mice and *Lcn2*$^{-/-}$ mice in the open field test to measure locomotor activity (10 min test duration). n = 8 mice per group. n.s. by unpaired Student's t-test. (**E**) No differences were observed between *Lcn2*$^{fl/fl}$ control littermates and *Cx3cr1*$^{CreER/+}$::*Lcn2*$^{fl/fl}$ mice in the accelerating rotarod test to measure motor function (5 min test duration). n = 8-10 mice per group. n.s. by unpaired Student's t-test. (**F**) No differences were observed between *Lcn2*$^{fl/fl}$ control littermates and *Cx3cr1*$^{CreER/+}$::*Lcn2*$^{fl/fl}$ mice in the open field test to measure locomotor activity (10 min test duration). n = 8-10 mice per group. n.s. by unpaired Student's t-test. (**G-J**) Representative traces and quantification of the frequency and amplitude of sEPSCs (**G** and **H**), as well as representative traces and quantification of the number of current step-induced AP firings (**I** and **J**) in spinal lamina IIo neurons from *Vglut2*$^{Cre/+}$::tdTomato mice treated with vehicle or LCN2 (1 μg, i.t., once per day for 3 consecutive days). n = 18-20 neurons from 4 mice per group. $^{**}p < 0.01$ by unpaired Student's t-test (**H**), $^{***}p < 0.001$ by two-way repeated ANOVA with *post hoc* Bonferroni test (**J**). (**K-N**)

Representative traces and quantification of the frequency and amplitude of sEPSCs (**K** and **L**), as well as representative traces and quantification of the number of current step-induced AP firings (**M** and **N**) in spinal lamina IIo neurons from *wt* mice and *Lcn2*$^{-/-}$ mice treated with GSK101 (1 μM, i.t., once per day for 3 consecutive days). n = 18 neurons from 4 mice per group. **p < 0.01, ***p < 0.001 by unpaired Student's t-test (**L**), ***p < 0.001 by two-way repeated ANOVA with *post hoc* Bonferroni test (N). (**O** and **P**) Representative images with 3D reconstruction and quantification of the dendritic spine density in spinal lamina IIo neurons from *Vglut2*$^{Cre/+}$::tdTomato mice treated with vehicle or LCN2 (1 μg, i.t., once per day for 3 consecutive days). n = 16-20 neurons from 4 mice per group. **p < 0.01 by unpaired Student's t-test. Scale bar = 10 μm. (**Q** and **R**) Representative images with 3D reconstruction and quantification of the dendritic spine density in spinal lamina IIo neurons from *wt* mice and *Lcn2*$^{-/-}$ mice treated with GSK101 (1 μM, i.t., once per day for 3 consecutive days). n = 12-13 neurons from 4 mice per group. ***p < 0.001 by unpaired Student's t-test. Scale bar = 10 μm. Data are represented as mean ± s.e.m.

Supplemental Table 1 Information for antibodies

Antibody	Manufacturer	Cat. No.
Mouse anti-NeuN	Millipore	Cat# MAB377; RRID: AB_2298772
Mouse anti-GFAP	CST	Cat# 3670; RRID: AB_561049
Rabbit anti-IBA1	Wako	Cat# 019-19741; RRID: AB_839504
Rat anti-CD31	Novus	Cat# NB100-1642; RRID: AB_10129871
Chicken anti-GFP	Aves Labs	Cat# GFP-1020; RRID: AB_10000240
Rat anti-LCN2	Abcam	Cat# ab70287; RRID: AB_2136473
APC anti-mouse CD45	Biolegend	Cat# 147707; RRID: AB_2563539
Brilliant Violet 510 anti-mouse CD11b	Biolegend	Cat# 101245; RRID: AB_2561390
Alexa Fluor 700 anti-mouse CX3CR1	Biolegend	Cat# 149035; RRID: AB_2629605
Brilliant Violet 421 anti-mouse CD192 (CCR2)	Biolegend	Cat# 150605; RRID: AB_2571913
Streptavidin, Alexa Fluor 488 conjugate	Invitrogen	Cat# S11223; RRID: AB_2336881
Streptavidin, Alexa Fluor 594 conjugate	Invitrogen	Cat# S32356; RRID: N/A
Donkey anti-Chicken secondary antibody, Alexa Fluor 488 conjugate	Jackson ImmunoResearch	Cat# 703-545-155; RRID: AB_2340375
Donkey anti-Rabbit secondary antibody, Alexa Fluor 488 conjugate	Invitrogen	Cat# A-21206; RRID: AB_2535792
Donkey anti-Rabbit secondary antibody, Alexa Fluor 594 conjugate	Invitrogen	Cat# A-21207; RRID: AB_141637
Donkey anti-Rabbit secondary antibody, Alexa Fluor 647 conjugate	Invitrogen	Cat# A-31573; RRID: AB_2536183
Donkey anti-Rat secondary antibody, Alexa Fluor 488 conjugate	Abcam	Cat# ab150153; RRID: AB_2737355
Donkey anti-Rat secondary antibody, Alexa Fluor 594 conjugate	Invitrogen	Cat# A-21209; RRID: AB_2535795
Donkey anti-Mouse secondary antibody, Alexa Fluor 594 conjugate	Invitrogen	Cat# A-21203; RRID: AB_141633

Supplemental Table 2 Primer sequences for RT-qPCR

Gene	Primer forward (5′-3′)	Primer reverse (5′-3′)	GenBank Accession
Lcn2	CCCCATCTCTGCTCACTGTC	TTTTTCTGGACCGCATTG	NM_008491
Gapdh	AAATGGTGAAGGTCGGTGTG	AGGTCAATGAAGGGGTCGTT	NM_008084
Tnf-α	GCCTCCCTCTCATCAGTTCT	ACTTGGTGGTTTGCTACGAC	NM_013693
Il-1β	GTACAAGGAGAACCAAGCAAC	CCGTCTTTCATTACACAGGA	NM_008361
Il-6	TAGTCCTTCCTACCCCAATTTCC	TTGGTCCTTAGCCACTCCTTC	NM_031168
CatS	GTTGGGCTTTCAGTGCTGTG	GGATAGGAAGCGTCTGCCTC	NM_001267695
Ccl2	TTAAAAACCTGGATCGGAACCAA	GCATTAGCTTCAGATTTACGGGT	NM_011333
Il-18	GACTCTTGCGTCAACTTCAAGG	CAGGCTGTCTTTTGTCAACGA	NM_008360
Bdnf	TCATACTTCGGTTGCATGAAGG	AGACCTCTCGAACCTGCCC	NM_001048141
